# Kidney Biopsy and Prognosis in Lupus Nephritis: Histological Predictors of Renal Outcome—A Narrative Review

**DOI:** 10.3390/medicina62091653

**Published:** 2026-08-28

**Authors:** Giovanni Maria Rossi, Chiara Pala, Francesco Fontana, Davide Gianfreda, Daniel Salvetti, Marco Delsante, Marco Allinovi, Domenico Giannese

**Affiliations:** 1 Nephrology Unit, Department of Medicine and Surgery, University Hospital of Parma, University of Parma, 43126 Parma, Italy; 2 Department of Pathology, Amsterdam University Medical Center, University of Amsterdam, 1105 AZ Amsterdam, The Netherlands; 3 Nephrology, Dialysis and Kidney Transplant Unit, University Hospital of Modena, 41124 Modena, Italy; 4 Nephrology and Dialysis Unit, Vito Fazzi Hospital, 73100 Lecce, Italy; 5 Nephrology, Dialysis and Transplantation Unit, Careggi University Hospital, 50134 Florence, Italy; 6 Nephrology, Dialysis and Transplantation Unit, Azienda Ospedaliero-Universitaria Pisana, 56124 Pisa, Italy

**Keywords:** lupus nephritis, kidney biopsy, ISN/RPS classification, activity index, chronicity index, tubulointerstitial lesions, thrombotic microangiopathy, antiphospholipid-antibody nephropathy, lupus podocytopathy, renal prognosis, kidney outcome, kidney failure, histopathology

## Abstract

The kidney biopsy is the cornerstone of prognostication in lupus nephritis, yet which features predict renal outcome—and which kind—remains incompletely defined. This narrative review relates eight histological axes to two families of renal outcome defined a priori: soft outcomes (renal flare/relapse and response/remission) and hard outcomes (doubling of serum creatinine, sustained decline in estimated glomerular filtration rate, and end-stage kidney disease; death is considered a competing, patient-level outcome). The axes span the ISN/RPS classification and its 2018 revision, individual glomerular and tubulointerstitial lesions, NIH activity and chronicity indices, lupus podocytopathy, lupus vasculopathy, antiphospholipid-antibody nephropathy, thrombotic microangiopathy and repeat biopsy. Across them, a consistent asymmetry emerges, although it is often not reproducible between studies. Chronicity—especially tubulointerstitial damage—predicts hard outcomes, whereas the activity index as a whole predicts response and flare. Cellular crescents and fibrinoid necrosis, active lesions that can signal hard outcomes, are the exceptions. Whether chronicity adds to baseline kidney function remains unsettled. Several prognostically important entities lie outside the proliferative classification; immune-deposit burden is largely diagnostic; and repeat histology adds information beyond the baseline biopsy. A recurring caveat is the therapeutic era, which modifies the very lesions scored.

## 1. Introduction

Lupus nephritis (LN) is among the most consequential manifestations of systemic lupus erythematosus (SLE), and the kidney biopsy remains the cornerstone of its assessment. Reading the biopsy for prognosis is not a new ambition: interest in how the histological appearance forecasts the renal course is essentially as old as the histological study of LN itself, and it is the thread that connects successive classifications. This review revisits that long-standing question with current data, asking which histological features predict renal outcomes—and, importantly, distinguishing the features that predict different kinds of renal outcome.

To set out the scale of the disease, LN develops in roughly one-third to one-half of patients with SLE over the disease course—38% in the prospective multi-ethnic SLICC inception cohort—and is more frequent and more severe in patients of African, Hispanic and East Asian ancestry than in those of European descent [1]. Among biopsied patients, the proliferative classes III and IV predominate (together accounting for roughly 55–70% of cases, class IV being the single most common type), pure membranous class V accounts for about 10–20%, mixed classes account for a further 5–15%, and classes I/II and VI account for the remainder [2]. Baseline scores are typically modest—activity-index medians are around 2–9 and chronicity-index medians are around 0–4 [3]. The entities that fall outside this framework are individually uncommon but collectively relevant: renal thrombotic microangiopathy (TMA) is reported in about 0.5–17% of biopsies, non-TMA vascular lesions in a substantial minority, and lupus podocytopathy in roughly 1–1.6% [4,5,6].

In this narrative review, we chose to separate renal outcomes into “soft” and “hard” outcomes, defined a priori and used throughout. Soft outcomes comprise renal flare or relapse and renal response or remission—the events that inform treatment changes. Hard outcomes comprise doubling of serum creatinine, a significant decline in estimated glomerular filtration rate (eGFR), end-stage kidney disease (ESKD) requiring dialysis or transplantation, and death—the irreversible endpoints that define long-term prognosis. The distinction matters because selected histological features might predict soft but not hard renal outcomes, and vice versa; conflating them has muddied the literature, since a lesion that drives relapse is not necessarily a lesion that drives ESKD.

A caveat on these hard endpoints is warranted. A graded ladder of surrogate endpoints for kidney-failure trials was validated over the past decade—from the legacy doubling of serum creatinine (equivalent to a 57% decline in eGFR) to a confirmed 40%, or even 30%, eGFR decline, and most recently the eGFR slope [7,8,9]. LN research has largely not migrated to these measures, so much of the cohort literature reviewed here still reports the now-somewhat-dated doubling of serum creatinine.

We examined eight histological axes in turn: the ISN/RPS classification and its revisions; individual glomerular and tubulointerstitial lesions; the NIH activity and chronicity indices; lupus podocytopathy; lupus vasculopathy other than TMA; antiphospholipid-antibody nephropathy (APSN); TMA; and the information added by repeat and per-protocol biopsy together with the clinical response. Two themes recur: an asymmetry between activity and chronicity, and the fact that several prognostically important entities lie outside the proliferative classification altogether. Because the underlying literature spans four decades, several successive classifications, heterogeneous scoring and divergent outcome definitions, we present a narrative review, with the structured but non-exhaustive search and expert, relevance-based study selection described in the Methods section.

## 2. Methods

We searched PubMed/MEDLINE from inception to 30 June 2026 for studies relating kidney histopathology to renal outcome in LN, combining three concept blocks—terms for LN, terms for kidney histopathology/biopsy/classification (including ISN/RPS class, the NIH activity and chronicity indices, and vascular/thrombotic-microangiopathic lesions) and terms for renal prognosis/outcome; the full search string is provided in the Supplementary Materials (Table S1). Because the eligible literature is markedly heterogeneous in design, histological scoring and outcome definition, we conducted a narrative rather than a systematic review; the search was structured and broad but not formally exhaustive. We included primary clinical studies of biopsy-proven LN, published in English, relating at least one histological feature to a renal outcome and excluded reviews, editorials and letters, case reports or series with fewer than ten patients, experimental studies, and marker-only studies without a histology-to-outcome correlation; non-English-language articles were likewise excluded during screening. Records were screened by title and abstract by a single reviewer through expert, relevance-based selection, without duplicate or independent screening, and were then assessed in full text. Single-reviewer, relevance-based selection is a deliberate feature of a narrative synthesis rather than a shortcoming of a systematic one, but it is a real limitation and we return to it below. A minimum sample-size criterion was applied and modulated by topic (≥100 patients for classification, the activity/chronicity indices and individual glomerular and tubulointerstitial lesions; ≥20 for the inherently smaller literature on vascular and thrombotic-microangiopathic lesions and on protocol/repeat-biopsy and flare studies).

**Figure 1:**
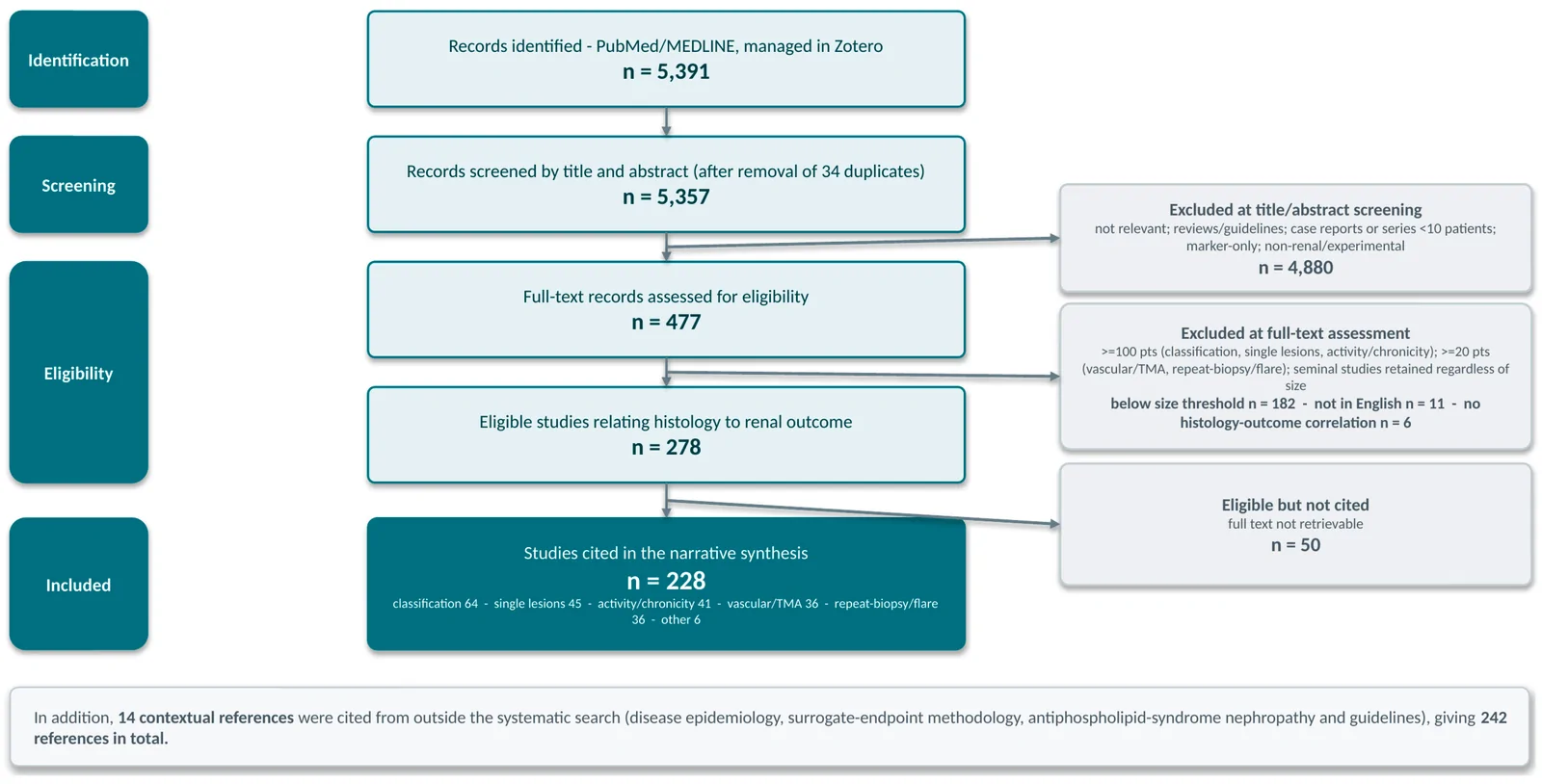
Flow of study identification, screening, eligibility and selection.

The search identified 5391 records (5357 after removal of 34 duplicates); 477 were assessed in full text. After exclusion of studies below the topic-specific size threshold (n = 182), non-English-language articles (n = 11), and studies whose prognostic analysis related only non-histological predictors to outcome without any histological variable in the model (n = 6), 278 studies relating at least one histological feature to renal outcome were eligible. Consistent with a narrative approach, we cited all eligible studies for which the full text could be obtained, together with a small number of seminal studies retained below the size threshold; 228 studies are cited in the synthesis (including a small number retained below the size threshold), whereas 50 eligible studies whose full text could not be retrieved are not cited. A further 14 references were cited for context (disease epidemiology, the validation of surrogate kidney-failure endpoints, and antiphospholipid-syndrome nephropathy and its guidelines), bringing the reference list to 242. The study-selection flow is summarized in Figure 1 for transparency. It is not a PRISMA diagram and should not be read as one: the selection it depicts was relevance-based and performed by a single reviewer, and no formal risk-of-bias assessment, quality scoring or quantitative synthesis was undertaken because the heterogeneity of scoring systems, outcome definitions and treatment eras across four decades of literature would not support them. In place of a formal appraisal instrument, Table 1 records for every representative cohort the outcome exactly as the source study defined it and whether the reported estimate was adjusted—the two characteristics that most determine how much weight an individual estimate can bear. For the same reason, we do not treat the number of studies on either side of a question as an argument in itself: where cohorts disagree we say so and indicate what distinguishes them. Findings are presented separately for soft and hard renal outcomes within each axis.

## 3. Histological Classification and Renal Outcome

The histological classification of LN is, before anything else, a prognostic statement. From the World Health Organization (WHO) categories to the 2003 International Society of Nephrology/Renal Pathology Society (ISN/RPS) system and its 2018 revision, each iteration has been assessed not only for how reproducibly it describes a biopsy but for how well the resulting class relates to renal outcome—a prognostic interest as old as the histological study of the disease itself. The evidence assembled below suggests that classification by lesion pattern captures a real prognostic signal—proliferative disease does worse—but that its independence and most of the durable, hard-outcome information depend on features the class label handles poorly: the balance of activity and chronicity, and the tubulointerstitial compartment.

### 3.1. Histological Class and Hard Renal Outcome

That proliferative classes carry the worst kidney survival is consistent across cohorts and classification systems. In the multicenter Italian GISNEL cohort of 659 patients, class IV conferred a hazard ratio of 2.0 for ESKD versus class III and 2.5 versus class V, with 10-year renal survival of about 80% [54]. In the long-term Japanese cohort of Yokoyama and colleagues, ESKD occurred in 40.9% of patients with class IV versus 2.6% of all other classes combined (*p* < 0.001); in multivariable Cox analysis, ISN/RPS 2003 class IV was the single strongest predictor (hazard ratio 14.8, 95% CI 1.4–155, *p* = 0.025—an estimate whose extreme imprecision reflects the very small number of events in the comparator arm and which should therefore be read as a direction of effect rather than as a magnitude), whereas the corresponding WHO 1995 class IV was not an independent predictor (hazard ratio 2.16, *p* = 0.57)—an early, direct demonstration that the 2003 system out-predicted its predecessor [10]. The same gradient has been reproduced across continents and decades, from early WHO-classified series to large modern registries: severe proliferative or sclerotic lesions consistently mark the poorest kidney survival [11,12,55,56,57,58,59]. In the large Chinese cohort of Tang and colleagues, a class IV or VI lesion carried a hazard ratio of 7.17 (95% CI 3.10–16.56) for ESKD, with every class VI patient progressing to kidney failure [11], and a cross-sectional Spanish registry of 1648 biopsies found that classes III/IV were independently associated with reduced eGFR already at the time of biopsy (OR 1.82, 95% CI 1.16–2.87) [58]. These hazard ratios come from different cohorts, eras and adjustment sets and are not adjusted for the same covariates, so their magnitudes are not directly comparable (Table 1). Large modern cohorts reinforce the magnitude of risk attached to proliferative disease while confirming that class is necessary but not sufficient for prognostication: outcome varies widely within any given class according to the balance of activity and chronicity and the burden of tubulointerstitial damage [31,60,61]. This dependence on co-factors rather than the label alone is now the dominant message of the contemporary literature [13,14,62,63].

That hard endpoints have become rarer as supportive and immunosuppressive care has improved is itself a recurring finding, and it tends to attenuate the apparent weight of class over time. In the half-century single-center experience of Moroni and colleagues (n = 499), ESKD fell from 24.8% to 1.3% and death from 19.8% to 3.6% across successive eras even though histological class and activity index were unchanged [14]; comparable secular improvements in remission and renal survival have been documented in Chinese, pediatric and other cohorts [13,64,65]. Across these series the durable predictors of ESKD are remarkably stable—baseline renal function, hypertension and the chronicity burden—rather than the class label as such [13,14,62,66,67].

An important and unresolved tension concerns how much of the prognostic weight of class IV survives multivariable adjustment. In several large or classic cohorts it does: class IV remained an independent predictor of ESKD with an odds ratio of 24.6 on univariable analysis, class IV remaining an independent predictor in the multivariable Cox model in a Hong Kong cohort [15] and around 5–15 in Latin American and admixed-race cohorts after adjustment for tubulointerstitial and chronicity scores [68,69]. The Saudi series of Al Arfaj and colleagues likewise retained both proliferative classes as independent predictors of ESKD (class III RR 7.704, class IV RR 2.939) [70], and class IV independently predicted all-cause mortality (HR 2.6) in the biopsy-proven cohort of Norby and colleagues [12]. In others, by contrast, WHO/ISN-RPS class loses independent significance once renal function, proteinuria, age and chronicity are entered [71,72]. This is especially evident in pediatric and developing-country cohorts, where aggressive immunosuppression appears to flatten the class gradient: neither childhood class IV nor WHO class predicted survival once clinical variables were modelled [67,73,74,75], and in one pediatric series renal and patient survival were indistinguishable between class II and class IV [73]. The reasonable reading is not that class is uninformative but that its independence is conditional on what else the model contains—and that, alone, it under-captures prognosis. Several of these cohorts make the point sharply by showing that what does survive adjustment is the clinical and chronic-lesion context: AKI or raised creatinine at presentation, hypertension, failure to remit and treatment non-adherence, rather than class [62,65,66,74,76].

The mortality attached to LN, finally, is largely uncoupled from the renal class and dominated by infection and cardiovascular events. Biopsy-proven LN carried a standardized mortality ratio of 5.6 versus the general population in the cohort of Norby and colleagues [12] and of about 3.6 in the Japanese series of Kono and colleagues [77], with infection and flare the leading causes of death across settings [11,65,74,76]. Older age at onset is a consistent independent predictor of both death and CKD: late-onset disease (≥50 years) predicted all-cause mortality with a hazard ratio of 3.03 despite milder active lesions [78], and across the pediatric-to-elderly age spectrum advanced age predicted CKD or death (RR 3.278) in parallel with a higher baseline chronicity index [63]. The role of male sex is more contested: several cohorts report worse renal and patient outcomes in men [57,73,77,79], yet in most the effect does not survive adjustment for the more advanced disease men present with [79], and a large international pooled analysis found no independent excess risk once baseline eGFR was accounted for [80].

### 3.2. The Class IV-S Versus IV-G Controversy

In the ISN/RPS system, class IV is subdivided according to the proportion of affected glomeruli showing segmental versus global lesions: IV-S (segmental) when more than half of the involved glomeruli have lesions affecting less than half of the glomerular tuft and IV-G (global) when more than half show lesions involving more than half of the tuft. No question in this literature has generated more data with less resolution than whether the segmental (IV-S) and global (IV-G) subclasses of diffuse proliferative nephritis differ in prognosis. The distinction has a respectable empirical origin: in the WHO-classified cohort of Schwartz and colleagues, prognosis was proportional to the extent of active segmental involvement—severe segmental glomerulonephritis (≥50% of glomeruli) carried a 5-year survival of about 59%, similar to diffuse proliferative disease (≈53%) and far worse than mild/moderate segmental disease (≈82%)—a finding that justified folding severe segmental injury into class IV [81,82].

Yet when the distinction was tested under the ISN/RPS definitions themselves, the prognostic difference largely evaporated. Single-center cohorts found no significant difference in hard outcomes between IV-S and IV-G: Mittal and colleagues reported comparable favorable-outcome rates (60% vs. 64%) and near-identical ESKD rates (10% vs. 9%) [83]; Hill and colleagues found 10-year survival free of creatinine doubling of roughly 65% versus 60% (*p* = 0.53) [84]; and in a large Chinese cohort renal survival did not differ significantly (*p* = 0.15), although ANCA positivity was higher in IV-S [85]. The meta-analysis of Haring and colleagues pooled eight studies and found a relative risk for creatinine doubling or ESKD of 1.08 (95% CI 0.68–1.70) for IV-G versus IV-S, with no heterogeneity [16]. The dissent comes mainly from Duque de Sá Carneiro Filho and colleagues, who found IV-G to carry higher activity and chronicity indices, more crescents and vascular lesions, and to independently predict CKD (eGFR < 60: OR 5.6, 95% CI 1.08–29.2), while arguing nonetheless that the two are stages of one disease and warrant identical treatment [86]. The most incisive explanation came from Schwartz and colleagues, who showed a definitional stage-migration effect: the most severe, widely distributed segmental cases are absorbed into IV-G by the ISN/RPS surface-area rule, simultaneously worsening the apparent IV-S group and diluting IV-G and thereby erasing a difference that is real at the level of pathology [87]. A complementary line of work bypasses the dichotomy altogether: unsupervised clustering of class III/IV biopsies by Bolognesi and colleagues resolved two reproducible morphological patterns—a membranoproliferative-like cluster with a higher activity index (10.52 vs. 5.72, *p* < 0.001) and a vasculitis-like cluster—that segregate the injury better than the IV-S/IV-G split and may carry pathogenetic meaning [88]. The practical conclusion, reflected in the 2018 revision’s decision to abandon the IV-S/IV-G subdivision [89], is that the segmental–global axis is biologically meaningful but that the ISN/RPS cut-off was the wrong instrument to capture it.

### 3.3. Activity, Chronicity, and the 2003-to-2018 Revision

If class and subclasses are imperfect predictors, the feature most consistently associated with hard renal outcomes across four decades is chronicity. The point was made at the outset by Austin and colleagues, whose NIH activity and chronicity indices showed a graded relationship with renal failure, with the chronicity index and tubular atrophy carrying the greatest weight and the combination of tubular atrophy, glomerulosclerosis and cellular crescents marking the highest-risk biopsies [90,91]. This primacy of the chronicity index over the activity index has since been reproduced in numerous independent cohorts spanning Asia, Europe and the Americas, in which the chronicity index—and tubular atrophy/interstitial fibrosis in particular—independently predicts ESKD or CKD, while the activity index does not [13,14,57,63,79,92,93,94]. In the half-century cohort of Moroni and colleagues, both indices were retained (activity index RR 1.1, chronicity index RR 1.29 per unit) [14], whereas in others, notably So and colleagues’ and Appel and colleagues’, neither index predicted hard outcomes once remission status was accounted for [93,95]. Within the ISN/RPS framework, Hiramatsu and colleagues showed that it was not the IV-S/IV-G label (*p* = 0.68) but the presence of chronic lesions that mattered: among class IV-G patients, none with purely active lesions reached creatinine doubling at 120 months versus 50% of those with combined active and chronic lesions (*p* = 0.029) [96]. The same active-plus-chronic signature predicted the renal endpoint independently (HR 9.07) in the long-term proliferative cohort of Ikeuchi and colleagues [97].

Three validation cohorts of the 2018 revision converge on the same message and, where tested head-to-head, favor the revised system. The 2018 revision recast the indices as a modified activity index (range 0–24) and chronicity index (range 0–12) applied to all ISN/RPS classes, lowered the crescent threshold to ≥10% of the glomerular tuft, refined the definitions of fibrinoid necrosis and crescents, and retained the double weighting of fibrinoid necrosis and cellular/fibrocellular crescents [89]. Tao and colleagues found that fibrous crescents (HR 4.10) and interstitial fibrosis/tubular atrophy exceeding 50% (HR 8.58), together with the modified chronicity index, independently predicted a composite of death, ESKD or ≥30% eGFR loss, whereas the original NIH indices did not [98]. In the only direct comparison, Umeda and colleagues showed that the 2018 modified chronicity index predicted a 30% eGFR decline (adjusted HR 1.32), while the 2003 class and its A/C subdivisions lost significance after adjustment [99]; Hachiya and colleagues reached the same conclusion, with the chronicity index (adjusted HR 1.24) and interstitial fibrosis (adjusted HR 2.66) predicting renal function decline, defined as a 1.5-fold rise in serum creatinine, while the 2003 class and subclass did not [100]. The independent prognostic weight of fibrous crescents specifically has been confirmed elsewhere, predicting ESKD with a hazard ratio of 3.439 in the poor-baseline-function cohort of Luo and colleagues and of 2.590 in the cohort of Wang and colleagues [79,101], and a high chronicity index above a cut-off of 4 segregated worse renal survival among class IV and IV + V patients in the random-survival-forest analysis of Wang and colleagues [94]. Severe tubular atrophy and interstitial fibrosis carried a hazard ratio of 7.05 for the adverse composite in the cohort of Zhang and colleagues, where it was the strongest pathological predictor [57], and crescents and interstitial fibrosis likewise predicted CKD in the large Egyptian series of Momtaz and colleagues [62].

A recurring observation in these modern cohorts is that the activity index, in contrast, is a weak predictor of hard outcomes—plausibly because active lesions are reversible under contemporary immunosuppression [92,93,95]. This generalization, however, requires an important qualification developed in the next sections: specific active lesions, namely, cellular/fibrocellular crescents and fibrinoid necrosis, can independently predict hard outcomes even when the composite activity index does not [62,79].

### 3.4. Class and Soft Outcomes

Class also informs response and relapse, though less sharply than it informs long-term function. In the Collaborative Study cohort, complete remission (serum creatinine ≤ 1.4 mg/dL with proteinuria ≤ 0.33 g/day) was more frequent in global/diffuse category IV than in segmental disease (60% vs. 38%) [82]. Across modern cohorts, however, the dominant determinant of soft outcomes is not the class label but achievement and timing of remission, with the strongest predictive value attaching to the response status reached at six to twenty-four months [75,93,101,102,103,104,105]. Failure to attain complete remission independently predicts both renal flare and progression to CKD or ESKD [93,101,103,105], early response is protective [75,102], and conversely renal response status—whether defined as proteinuric remission, complete renal response, or the BLISS-LN-derived mPERR target—carries durable prognostic weight: in the cohort of Cooper Blenkinsopp and colleagues, mPERR at two years predicted freedom from chronic renal insufficiency (adjusted HR 0.26) [102], and in the incident cohort of Pirson and colleagues failure to remit raised the risk of CKD roughly threefold and that of ESKD roughly tenfold [103]. The predictors of remission itself are clinical rather than histological—chiefly baseline renal function and proteinuria—with class generally non-predictive once these are modelled [67,104,106,107,108,109,110]. In the Portuguese cohort of Farinha and colleagues, an eGFR ≤ 75 mL/min/1.73 m^2^ at one year was the single strongest predictor of progression to CKD (HR 22.86), while proteinuria at diagnosis predicted neither class nor long-term CKD [111], and lower baseline proteinuria predicted complete renal response in several independent series [106,107,111]; even low-level baseline proteinuria, however, frequently accompanies proliferative LN and still carries substantial long-term risk [112].

Membranous and mixed classes display a distinct relapse behavior taken up below, but the prognostic standing of coexisting membranous features is already apparent at the level of response and hard outcome. Mixed proliferative-and-membranous disease (class III/IV + V) predicted the renal endpoint in the cohort of Ikeuchi and colleagues, with a univariable hazard ratio of 6.06 attenuating to 2.13 on multivariable adjustment [97]; the signal is not universal, however, since Ilori and colleagues found no association between mixed versus pure proliferative histology and either short-term ESKD or complete remission [113]. Relapse, finally, is governed mainly by the depth of the preceding remission and by maintenance strategy rather than by class: partial rather than complete remission, glucocorticoid-only or azathioprine-based maintenance, younger age and anti-RNP or anti-SSB positivity recur as risk factors across cohorts [105,107,110,114,115,116,117]. In class I/II disease, glucocorticoid monotherapy was an independent risk factor for renal relapse (HR 2.71) and clustered all observed ESKD events [114], and azathioprine maintenance carried a nearly fivefold higher flare rate than mycophenolate (HR 4.78) in the Hispanic cohort of Mejía-Vilet and colleagues [105], underscoring that for the softer outcomes the modifiable clinical course outweighs the static histological class.

## 4. Individual Histological Lesions and Renal Outcome

A classification reduces a biopsy to a category; in practice, much of its prognostic content is carried by individual lesions whose weight is unequal and, importantly, not confined to the glomerulus. Studies that score lesions separately rather than collapsing them into a class converge on three messages: a small number of glomerular lesions are genuinely adverse, the tubulointerstitial compartment is at least as important as the glomerulus for hard outcomes, and the immune-deposit burden carries little prognostic information except where its location or type is unusual.

### 4.1. Active Glomerular Lesions: Crescents and Fibrinoid Necrosis

Among glomerular lesions, fibrinoid necrosis and crescents are the ones that recur as independent predictors, and they are also the principal exception to the rule that activity is prognostically weak. In the Leiden–Rotterdam cohort, fibrinoid necrosis predicted both renal flare and ESKD (HR 1.08 per percent of glomeruli involved), while fibrous crescents independently predicted ESKD [18]. Crescents carried a similar weight in a Romanian cohort (HR 1.07 per percent) [118], and a graded relationship between the extent of crescents and ESKD was demonstrated in a 520-patient diffuse-proliferative cohort [119]. Several recent, adequately adjusted studies show that this signal survives multivariable analysis: the proportion of crescents independently predicted progression and mortality after adjustment for the activity and chronicity indices and clinical variables [19]; cellular and fibrocellular crescents were independent predictors of creatinine doubling or ESKD [20,120]; and fibrinoid necrosis predicted a shorter time to ESKD (HR 4.09) alongside the chronicity index [121]. A consistent body of work confirms that overtly crescentic lupus nephritis is a distinct, higher-risk phenotype: crescentic disease accounts for roughly a fifth of class IV-G biopsies and conferred poorer long-term renal survival even after adjustment for baseline creatinine and the chronicity index [122], and where crescents define the lesion the burden becomes quantitatively graded, with patients exceeding 50% crescents at markedly elevated ESKD risk (HR 2.78) and almost 90% of those with rapidly progressive presentation and the heaviest crescent loads reaching ESKD [123]. Notably, in these severe series it is the baseline serum creatinine rather than the crescent percentage itself that emerges as the dominant independent predictor, following an S-shaped relationship with ESKD probability [123], and the prognostic weight of crescents may be greatest in patients with lower-grade proteinuria (a significant crescent-by-proteinuria interaction, HR 2.68 below 3.5 g/24 h) [120]. The same picture recurs when severe disease is defined clinically rather than morphologically: acute kidney injury at presentation is common (occurring in about a fifth of patients) and an independent risk factor for ESKD or creatinine doubling (HR 5.82), with crescentic glomerulonephritis and thrombotic microangiopathy carrying the worst renal prognosis among AKI phenotypes [124]. The lesson is not that the activity index as a whole predicts hard outcomes—it generally does not—but that its crescent and necrosis components specifically can; indeed, in several cohorts the activity index was non-predictive, while crescent extent and the chronicity index were not [123,125]. The mechanistic correlate of this crescentic burden is beginning to emerge, with glomerular mTORC1 pathway activation tracking the percentage of cellular–fibrocellular crescents and independently predicting a composite of death, ESKD or eGFR decline (adjusted HR 3.36) [126]. By contrast, other apparently active glomerular features behave more benignly: the wire-loop lesion in class III/IV disease marked serological activity (higher anti-dsDNA, lower C3) but was not an independent predictor of CKD once tubular atrophy, baseline eGFR and chronicity were accounted for [127], echoing the counter-intuitive but consistent observation that several active endocapillary lesions behave as reversible, treatment-responsive features associated with eGFR recovery rather than loss [18]; consistent with this, in a trial setting, glomerular monocyte infiltration—an active, potentially reversible feature—predicted treatment response [128].

### 4.2. The Weight of the Tubulointerstitium

The strongest single-lesion signal for hard outcomes is tubulointerstitial. In the Dutch cohort, interstitial fibrosis and tubular atrophy of at least 25% conferred a hazard ratio of 3.89 for ESKD and, in the longitudinal model, the largest effect on eGFR of any histological variable [18]. The finding is highly reproducible: interstitial fibrosis and tubular atrophy mapped onto the ISN/RPS classes independently predicted doubling of creatinine or ESKD (interstitial infiltration HR 1.85, tubular atrophy HR 2.35, interstitial fibrosis HR 1.96) in a 313-patient Chinese cohort [21]; interstitial fibrosis was the only independent histological predictor of ESKD (HR 3.2) in a Taiwanese cohort [129]; moderate-to-severe interstitial fibrosis and tubular atrophy predicted ESKD (HR 5.18, and 6.75 within proliferative disease) and death independent of ISN/RPS class in a 25-year US cohort [22]; tubular atrophy (HR 2.28) and tubulointerstitial inflammation (HR 3.13) predicted kidney failure in a 526-patient cohort [23]; and in an admixed Brazilian cohort the chronicity index was the only independent predictor of kidney replacement therapy [130]. The tubular compartment carries weight even when formal atrophy is minimal: a structured tubular histological grade was a strong independent predictor of renal survival (grade 2 RR 31.2), and tubular expression of ICAM-1 and CD40 added prognostic information beyond the WHO class [131]. Tubulointerstitial inflammation matters too, with several refinements: interstitial inflammation, graded as a variable in its own right, independently predicted renal survival [24], tubulitis was the heaviest individual predictor in the Romanian series (HR 13.1) [118], and total cortical interstitial inflammation—scored across scarred as well as unscarred cortex—predicted progression (HR 2.45) where the conventional NIH score, restricted to unscarred cortex, did not [132]. The independence of the inflammatory signal from the fibrotic one is now well documented: the extent of tubulointerstitial inflammation predicted renal survival (HR 4.9) after adjustment for IF/TA grade and comorbidity [133], and tubulitis specifically retained an independent effect on eGFR over and above the ISN/RPS class in serial-biopsy data (coefficient −17.63), where moderate-to-severe tubulointerstitial lesions rose from 25% of first biopsies to 38% at repeat [134]. Tubular macrophages and epithelial cells within tubular lumina were among the strongest predictors in the French protocol-biopsy cohort [135]. That severe tubulointerstitial injury can dominate the prognosis is shown even in otherwise low-risk settings—in pure membranous lupus nephropathy, a severe tubular-interstitial lesion was an independent risk factor for ESKD (HR 72.2; an unstable estimate arising from a small number of events, and to be read as direction rather than magnitude) despite excellent overall renal survival [136]—and the same compartment underlies the worse chronicity seen in late-onset disease, where milder active glomerular lesions are accompanied by more severe tubular atrophy and interstitial fibrosis, even when the long-term outcome does not differ from that of early-onset patients [137]. Achieving and, crucially, sustaining complete renal response mitigates this burden, with the duration of sustained response (and persistent IF/TA) independently shaping eGFR decline and the ESKD/death composite [138,139]. The foundational demonstration that the biopsy adds prognostic information beyond clinical data, driven by glomerular scarring rather than the WHO class, dates to Whiting-O’Keefe and colleagues, and the message—that the tubulointerstitium deserves explicit, quantitative scoring—is now embedded in the 2018 chronicity index [140,141].

### 4.3. Immune Deposits: Usually Diagnostic, Occasionally Prognostic

Two findings refine the picture. First, the glomerular immune-deposit burden is, in general, a poor prognosticator: neither immunoglobulin intensities nor a full-house pattern predicted any outcome in the Dutch cohort, and total immunoglobulin deposits were non-predictive in the French cohort, in which only C3 retained prognostic value [18,135]. This blanket conclusion is reinforced at scale and across compartments: renal C1q and C3 deposits correlated with unfavorable clinicopathological features but did not predict ESKD or death in a 183-patient cohort [142], complement deposition by intensity carried no independent weight, and even “scanty” immune-deposit disease—milder proliferative lesions and a lower activity index—showed renal and patient survival indistinguishable from immune-complex-rich lupus nephritis [143]. Deposits define the class but do not, as a rule, forecast its course. This blanket statement, however, needs several qualifications based on deposit location and type. Immune deposits along the tubular basement membrane are associated with worse renal outcome (HR 4.2) [115], although in adequately adjusted analyses this signal does not always survive multivariable testing and is most evident in non-proliferative disease [115]. Prominent IgA deposition marks milder disease with a protective effect [144], as does exostosin positivity in membranous LN [145]; conversely, intense glomerular IgM deposition was the single immunoglobulin class independently associated with creatinine doubling or ESKD (adjusted HR 1.49), outperforming IgG, IgA, C1q and C3, though it did not predict mortality [146]. The pattern of complement activation may matter more than its mere presence: glomerular activation restricted to the alternative pathway (C3 without C1q/C4), seen in roughly a fifth of patients, predicted progression independently of the chronicity index (adjusted HR 3.49) despite lower overall disease activity [125]. The histological company that deposits keep also refines risk: ANCA positivity in lupus nephritis aggregates with distinct morphology—class IV-S, glomerular necrosis, segmental endocapillary hypercellularity and a higher crescent burden—and more active serology, yet does not independently worsen renal outcome after adjustment for severity and treatment [147], even though it may flag excess mortality within the scanty-deposit subgroup [143]. Second, chronic lesions are not entirely fixed: in serial biopsies, interstitial fibrosis regressed in a substantial minority of patients, and such regression carried an excellent prognosis [135]. Finally, the relevance of the biopsy is not confined to deposit-defined or proliferative lesions: even hematological features such as thrombocytopenia, though associated with higher histological activity, did not by themselves alter renal or patient prognosis, with eGFR and anemia remaining the operative predictors within that subgroup [148]; and decisions as concrete as glucocorticoid withdrawal track clinical rather than deposit variables, with nephrotic-range disease at onset predicting an inability to taper [23]. The lesion-level evidence therefore reinforces the central theme—necrosis, crescents and, above all, tubulointerstitial scarring drive hard outcomes, whereas deposit-defined features are largely reversible or prognostically silent [149].

## 5. The NIH Activity and Chronicity Indices

The 2018 revision did not only abandon the IV-S/IV-G subclass; it folded the NIH activity index (AI) and chronicity index (CI) into the classification itself, making them the formal instrument for the activity-versus-chronicity distinction invoked repeatedly in the preceding sections. Introduced by Austin and colleagues from the prospective NIH trials, the AI (range 0–24) aggregates six active glomerular and interstitial lesions—endocapillary hypercellularity, leukocyte infiltration, subendothelial hyaline deposits, fibrinoid necrosis/karyorrhexis and cellular crescents (the latter two double-weighted), and interstitial inflammation—while the CI (range 0–12) sums glomerulosclerosis, fibrous crescents, tubular atrophy and interstitial fibrosis [91,150]. The same group’s foundational analysis of severe lupus nephritis had already shown that adding histology—in particular the combination of cellular crescents and moderate-to-severe interstitial fibrosis—to a clinical model of serum creatinine, hematocrit and race significantly improved prediction of creatinine doubling and that an activity index above 7 together with a chronicity index above 3 marked the highest-risk biopsies [151]. Four decades of cohorts have asked whether these composites predict renal outcome, and the answer has settled into a clear asymmetry: chronicity predicts hard outcomes reliably, activity does so only in specific contexts.

### 5.1. The Chronicity Index Predicts Hard Renal Outcome

The most consistent signal in this literature is that a high chronicity index marks a kidney already on a downward trajectory. In a single-region Italian cohort of 203 patients followed for a median of 14 years, a baseline chronicity index above the median was associated with a sustained ≥30% loss of renal function, and its components were adverse in multivariable models, most consistently fibrous crescents (OR 5.18, 95% CI 2.43–11.04, *p* < 0.001) and glomerulosclerosis (OR 2.12, 95% CI 1.00–4.50, *p* = 0.05), with moderate-to-severe tubular atrophy (OR 3.17, 95% CI 1.04–9.64, *p* = 0.04) and interstitial fibrosis (OR 2.36, 95% CI 1.05–5.32, *p* = 0.04) reaching significance in the histological models [152]. The same group’s earlier work had already shown the chronicity index, baseline creatinine and antiphospholipid positivity to be the independent predictors of long-term chronic renal insufficiency, with the activity index non-predictive [38]. Across cohorts, the per-point hazard of the chronicity index for hard outcomes is remarkably stable—around 1.2 to 1.45 [26,29,100]—and a modern Accelerating Medicines Partnership cohort found it to be the single strongest predictor of sustained eGFR decline or ESKD (HR 1.33 per unit), with the activity index and ISN/RPS class non-predictive [28]. The synthesis by Choi and colleagues traces the same thread across earlier and modern series [150].

This per-point hazard recurs with striking constancy across geographically and methodologically diverse cohorts, anchoring the chronicity index as the most reproducible histological predictor of hard renal outcome [32,153,154,155,156]. In one of the earliest demonstrations, a chronicity index above 3 was the only independent histologic predictor of decreased renal survival, with WHO class alone non-predictive [153]. In proliferative disease, each one-unit increment in the chronicity index raised the odds of progression to ESKD or death (OR 1.77 per unit), whereas the activity index was non-significant [32]; comparable per-unit hazards have been reported for ESKD (OR 1.48) [154], for ESKD even among patients with minimal proteinuria (HR 1.48) [155], and for the composite of doubling creatinine, ESKD or death (HR 1.40) [156]. A large multivariate analysis confirmed the chronicity index as an independent histological term for the composite hard endpoint (HR 1.35 per unit) alongside cellular crescents (HR 1.44) [30]. Stratified analyses sharpen the dose response: relative to a low index, a moderate chronicity index (3–5) carried a sixfold and a high index (6–12) a twentyfold hazard of ESKD or death [33]. The chronicity index also tracks with mortality, predicting all-cause death in a 1048-patient cohort (HR 1.18) [157] and patient survival in a separate series (RR 1.54) [158]. The signal is reinforced where higher chronicity scores cluster with the worst outcomes—late-onset patients, who carried the highest chronicity scores, showed the greatest progression to CKD and the highest mortality [159]—and where individual chronic components recur as independent risks, as for the tubular atrophy score in a Chinese cohort (HR 1.25) [160]. The practical message is that chronic glomerular and, crucially, tubulointerstitial damage already present at biopsy is largely irreversible and sets a ceiling on recoverable function. A high chronicity index also predicts failure to achieve a renal response [161,162], a relationship confirmed by independent series in which a high or rising chronicity index lowered the probability of complete remission or treatment response [163,164,165,166]; in proliferative disease, a low chronicity index was the sole independent predictor of complete response at 12 months [164], a chronicity index above 2 independently predicted failure to achieve complete response [165], and a high total chronicity index independently predicted non-remission within a combined clinico-histological model reaching excellent discrimination (AUC 0.924) [163].

### 5.2. The Activity Index: Weak Overall but Driven by Specific Lesions

The activity index behaves very differently. In the Italian cohort, a baseline AI above the median did not predict functional loss (OR 1.68, 95% CI 0.77–3.68); the only active component that carried weight was interstitial inflammation, and only when it coexisted with chronic tubulointerstitial lesions [152]. The usual interpretation—that active lesions are the treatable and reversible component—is supported by the modern revision cohorts in which the composite activity index is repeatedly non-predictive of hard outcomes; multivariable analyses adjusting for age and creatinine confirm that the activity index, unlike the chronicity index, is not associated with the hard composite endpoint [32,33]. Three qualifications matter. First, in cohorts with unusually severe active disease the activity index above an extreme cut-off does predict ESKD [167], and in some adjusted analyses a modestly elevated activity index retains independent weight (OR 1.14; HR 1.17) [121,168]; consistent with this, an activity index above 11 was associated with reduced renal survival on univariate analysis in severe disease [153], and a higher activity index independently predicted hard outcome in one matched series (HR 1.30) [156]. Second, and most important, this independent signal is carried by particular active lesions—cellular and fibrocellular crescents and fibrinoid necrosis (Section 4.1)—rather than by the index as a whole; cellular and fibrous crescents themselves carried large independent hazards for ESKD (HR 4.42 and 5.93, respectively) [156], and cellular crescents independently predicted the composite hard endpoint [30] and adversely predicted remission [163]. Paradoxically, where activity reflects treatable inflammation a higher activity index can predict better short-term response in well-treated patients, while in other series a higher activity index reduced the likelihood of complete response, underscoring that the prognostic meaning of activity is context-dependent [156,165,169]. Third, activity regains prognostic value at repeat biopsy: in a re-biopsy subset, both a persisting activity index and a chronicity index at the second biopsy independently predicted functional loss because activity that survives treatment signals refractory disease rather than reversible inflammation [44]. Activity at presentation is therefore a statement about what therapy might reverse; activity that remains is a statement about what it has not.

### 5.3. From Indices to Validated Prognostic Scores

Because individual lesions carry uneven weight, several groups have distilled them into prognostic scores. Kojo and colleagues derived a weighted score dominated by fibrous crescents and glomerulosclerosis that discriminated renal insufficiency with an area under the curve of 0.794 and outperformed the IV-S/IV-G subclassification [170]. Tang and colleagues built and externally validated an integer score combining clinical variables with cellular and fibrous crescents, glomerulosclerosis, interstitial fibrosis and both indices; it stratified the five-year risk of ESKD from 2.7% to 79% [167]. In long-term, externally validated bedside models, the chronicity index recurs as the dominant histological term [26,171]. The same logic of supplementing histology with the clinical context recurs across cohorts, in which baseline serum creatinine, hypertension, anemia and disease activity scores add independent prognostic weight to the biopsy reading [30,166,172,173]; hypertension at onset, in particular, emerged as an independent predictor of mortality, ESKD and creatinine doubling and clustered with higher AI/CI scores [173], while combined clinico-histological models confirmed that the predictive yield of the biopsy is maximized when read alongside clinical variables [163,174]. These tools confirm that the prognostic information of the biopsy is best captured not by the class label but by a weighted reading of activity and, above all, chronicity.

### 5.4. Reproducibility and Structural Critique of the Indices

Two caveats temper enthusiasm. The indices are only moderately reproducible—reliability coefficients of around 0.48 for the AI and 0.57 for the CI [150]. And their internal structure has been questioned: in a two-cohort characterization of 194 biopsies, the activity index never approached its theoretical ceiling and applied poorly, by construction, to classes I, II and V (which largely lack the active lesions it scores), while factor analysis argued against the double-weighting of fibrinoid necrosis and cellular crescents and against placing fibrous crescents in the chronicity score [3]; a separate re-evaluation concluded that segmental glomerulosclerosis should be removed from the chronicity index without loss of predictive yield [27]. The dependence of the activity index on the presence of proliferative lesions is borne out in pure membranous disease, where the chronicity index nonetheless retained independent prognostic value for chronic renal insufficiency [172], and the limited yield of the active glomerular score is further illustrated where clinical and serological markers that correlate strongly with the renal activity index—anti-C1q antibodies and serum C1q—nonetheless failed to predict long-term renal outcome [175]. The indices’ performance is also modulated by features they do not capture directly, such as ANCA positivity, which associates with higher chronicity indices, more glomerulosclerosis and worse renal survival [158,176], and hyperuricemia, which clusters with higher activity and chronicity scores and independently predicts progression to CKD [177]. Taken together with the validation data, these analyses support the current direction—a streamlined, chronicity-weighted scoring applied across all classes—rather than abandonment of the indices, which remain the most direct histological predictors of renal outcome available.

## 6. Lupus Podocytopathy

Lupus podocytopathy is the clearest example of a biopsy diagnosis that falls outside the ISN/RPS proliferative framework yet carries its own distinct prognosis. It accounts for roughly 1 to 1.6% of LN biopsies and is defined by nephrotic syndrome with diffuse foot-process effacement on electron microscopy in the absence of peripheral capillary-wall immune deposits, on a light-microscopic background of minimal change, mesangial proliferation or focal segmental glomerulosclerosis (FSGS) [6,178]. Because the entity is defined podocyte-first rather than by immune-complex deposition, its outcome is governed less by the activity/chronicity axis than by steroid responsiveness, relapse and the FSGS subtype.

### 6.1. Soft Outcome: High Responsiveness, High Relapse

The dominant outcome message is favorable induction with troublesome relapse. In the foundational cohort of 50 patients, all presented with nephrotic syndrome and 94% achieved remission at a median of four weeks, but renal relapse occurred in 60% over a median follow-up of 62 months [6]. A larger cohort of 98 patients reproduced this pattern—complete renal response in 79% within twelve weeks, with no significant difference between glucocorticoid monotherapy and combination therapy, but relapse in 60% over a median of 78 months—and identified failure to achieve a complete renal response within twelve weeks, maintenance with glucocorticoids alone, and acute kidney injury at onset as independent risk factors for relapse [179]. The practical implication, echoing the proliferative literature, is that monotherapy controls the initial episode but that the addition of immunosuppressants restrains the high relapse rate.

### 6.2. Hard Outcome and the FSGS Subtype

Hard renal outcomes are, in aggregate, reassuring but subtype-dependent. Across both major cohorts, no patient reached ESKD or died over five to seven years of follow-up [6,179]. The risk, when present, tracks with the FSGS pattern: compared with minimal-change or mesangial patterns, this subtype shows a significantly higher incidence of acute kidney injury, more severe tubulointerstitial injury, higher chronicity and a markedly lower complete-response rate [6,179]. Acute kidney injury at presentation was itself predicted by heavy proteinuria and low C3, and pathological transition to a proliferative or sclerosing pattern can follow repeated relapses [178]. Lupus podocytopathy thus behaves prognostically like a podocyte disease grafted onto lupus: generally good kidney survival, but with the focal-segmental variant and recurrent relapse marking the patients in whom chronic damage accumulates.

## 7. Lupus Vasculopathy Other than Thrombotic Microangiopathy

Beyond TMA, the renal vessels in LN can show a spectrum of lesions that the ISN/RPS class does not capture: uncomplicated vascular immune-complex deposits, non-inflammatory necrotizing (lupus) vasculopathy, true renal vasculitis and arterio-arteriolosclerosis [36,180]. These lesions are common—vascular involvement of some kind was present in roughly three-quarters of biopsies in single-center series (74.6% and 81.8% in two Chinese series; [180,181])—yet their prognostic meaning has been obscured by decades of inconsistent nomenclature. Larger and more recent cohorts confirm both their frequency and their adverse weight: in a 225-patient series, renal vascular lesions were present in 44.9% of biopsies (12.9% moderate-to-severe), were more common in proliferative disease, and remained an independent risk factor for the composite of death, ESKD or a ≥30% rise in creatinine (adjusted HR 1.920, 95% CI 1.011–3.645) [35]. Disentangling them is worthwhile because they do not all carry the same risk.

### 7.1. Vascular Immune-Complex Deposits and Lupus Vasculopathy: Not Benign

The non-TMA lesion with the most consistent adverse signal is the immune-deposit-rich, non-inflammatory occlusive lesion historically termed lupus vasculopathy. In the multicenter Italian GISNEL cohort, renal vascular lesions overall conferred a hazard ratio of 5.49 for end-stage failure, with a five-year renal survival of 74% versus 90%; the immune-type lupus vasculopathy subgroup had a five-year renal survival of 68%, while arteriosclerosis was prognostically neutral [34]. Modern series that classify the glomerular disease by ISN/RPS while scoring the vascular lesions separately confirm that the deposits themselves are adverse: in a 341-patient Chinese cohort, vascular immune-complex deposits were associated with the renal endpoint on univariable analysis (HR 3.38; not independent after multivariable adjustment), an outcome poorer than every other vascular lesion except TMA [180], and a 740-patient cross-sectional re-appraisal documented the same distribution of vascular lesions, although without outcome data [182]. A complementary line of evidence comes from complement footprinting of the vessel wall: C4d deposition is virtually ubiquitous in LN, but its arteriolar localization tracks specifically with renal microvascular lesions and worse prognosis, the arteriolar co-deposition of C4d and C3c emerging as an independent predictor of ESKD or doubling of creatinine (HR 3.681, 95% CI 1.519–8.921) [183]. This reframes a feature long regarded as incidental into an independent marker of risk.

### 7.2. Necrotizing Vasculopathy and True Vasculitis: Severe but Not Independent

Non-inflammatory necrotizing vasculopathy behaves differently. It is reliably a marker of active, proliferative, complement-consuming disease, but in the cohorts that tested it formally it was not an independent predictor of renal outcome (HR 0.25, *p* = 0.33; HR 1.06, *p* = 0.96), its apparent severity being attributable to the proliferative and tubulointerstitial disease it accompanies [180,181]. The picture may differ in childhood-onset disease: in a pediatric cohort in which renal vascular lesions occurred in about 22.6% of biopsies, non-inflammatory necrotizing vasculopathy was specifically associated with a worse long-term composite outcome (HR 7.08, 95% CI 1.67–30.03) apparently independent of LN class and activity, whereas the presence of any vascular lesion showed only a trend (HR 3.45, 95% CI 0.86–13.80) [37]—a dissociation that may reflect the lower burden of competing chronic damage in younger patients. True renal vasculitis is genuinely severe but rare, concentrated in active class IV disease and present in numbers too small for reliable outcome estimates [34,184].

### 7.3. Folding Vascular Lesions into the Classification and the Toronto Caveat

The practical question is whether scoring these lesions adds prognostic information to the ISN/RPS framework. Incorporating chronic vascular lesions into a vascular-augmented chronicity index improved outcome prediction relative to the classical index (hazard ratio 2.32 versus 2.03) [180], and in a 429-patient cohort modeling the full vascular spectrum no single vascular lesion remained independent once activity and chronicity scores were included; in the model that excluded the chronicity score, chronic TMA, arteriosclerosis and true renal vasculitis all retained significance [36]. The prospective Toronto cohort tempers this: renal vascular lesions—predominantly arteriosclerosis—were associated with hypertension, lower eGFR and a higher chronicity index at biopsy and with subsequent systemic arterial events, but were not independently associated with chronic kidney disease, ESKD or death (the study being underpowered for renal endpoints) [185]. The neutral prognostic standing of arteriosclerosis in particular is reproduced in other series: in a 79-patient Chinese cohort, renal arteriosclerosis was common and clustered with vascular immune deposits, hypertension and worse cardiac structure and function, yet it was not an independent predictor of the combined hard endpoint (univariate HR 1.548, 95% CI 0.415–5.778), serum creatinine and left-ventricular dimension being the independent risks instead [186]. The downstream functional correlate of accumulated vascular and tubulointerstitial damage may even be captured non-invasively: a high renal resistive index (RRI > 0.7, concentrated in class IV) correlated strongly with the chronicity index and, together with chronicity and age, independently predicted non-response to induction therapy [187]. The emerging position is not that vascular lesions deserve a separate class, but that the immune-deposit and chronic vascular components carry real, partly independent prognostic weight that the current indices under-record, with non-inflammatory necrotizing vasculopathy best understood—at least in adults—as a marker of disease activity rather than an independent determinant of outcome.

## 8. Antiphospholipid Syndrome Nephropathy

Unlike the axes considered so far, the APSN literature is almost entirely oriented to hard outcomes. Soft outcomes—renal flare and response to induction—are rarely reported for this lesion, in part because APSN is identified retrospectively on biopsies performed for other indications and in part because it has no established lesion-specific treatment whose effect on response could be measured. The soft-outcome column is therefore largely empty here, and we say so rather than infer it.

APSN is the renal expression of antiphospholipid-mediated vascular injury; histologically, it overlaps with TMA but extends to a distinct set of chronic vaso-occlusive lesions. First characterized in primary antiphospholipid syndrome and then in lupus, it comprises an acute lesion—TMA—alongside fibrous intimal hyperplasia of interlobular arteries, organized arterial and arteriolar thrombi with or without recanalization, fibrous arteriolar occlusions, and the characteristic focal cortical atrophy, the renal analogue of the ischemic lesions seen elsewhere in the syndrome [188,189,190]. A defining feature on immunofluorescence is the absence of immune deposits within these vascular lesions, distinguishing them from immune-complex disease.

Among lupus biopsies, these lesions are far from rare: they were present in about a third of unselected lupus biopsies in the founding series and in roughly 40% of antiphospholipid-antibody-positive patients versus only 4% of antibody-negative patients [189,191]. The association with the antibody profile is consistent in direction but variable in detail—lupus anticoagulant in one series, anticardiolipin and triple positivity in another—reflecting differing cohorts and definitions [39,189,191]. Independent series reinforce the antibody association while also illustrating its variability: in 79 biopsies APSN was found in 11.4% and was significantly more frequent with the combination of lupus anticoagulant and anticardiolipin IgG (OR 3.61, 95% CI 1.28–5.14), the APSN patients carrying a higher chronicity index and more chronic lesions [192].

Prognostically, APSN is reliably associated with hypertension and a raised serum creatinine at biopsy, independent of the coexisting LN, and antiphospholipid positivity is itself an independent predictor of long-term chronic renal insufficiency (odds/hazard ratio ≈ 2.2) [38,189]. The clinical hazard attached to the antibody is again borne out: in a 49-patient series, antiphospholipid seroprevalence of 41% identified patients with substantially more thrombotic events (40% versus 10.4%) and a tendency toward higher CKD prevalence, although in that cohort the independent predictors of an eGFR below 60 were age, baseline eGFR and hypertension rather than the antibody itself [193]. Its weight regarding hard outcomes remains contested: one histological cohort found a strong independent association with progression to ESKD (odds ratio 5.8, 95% CI 1.7–19.7), with intimal changes and tubular atrophy as the heaviest correlates [39], whereas others—including the APSN series above, in which established renal damage at last follow-up did not differ from that of aPL-negative patients (33.3% versus 21.4%) [192]—found no significant excess of ESKD or death despite histological progression on serial biopsy [191,194]. No large prospective study with adjusted hazard ratios exists, and the evidence base is uniformly retrospective and modest in size.

Like the other vascular entities, APSN is not captured by the ISN/RPS classification—the 2018 revision again deferred formal vascular scoring to future work—even though incorporating chronic vascular lesions into the chronicity index improves outcome prediction [89,180]. Its principal practical implication is therapeutic: recognizing it shifts the balance from immunosuppression toward antithrombotic/antiplatelet and RAAS-blockade treatment, since the thrombotic lesions do not respond to immunosuppression alone—although in secondary, lupus-associated antiphospholipid-related TMA there is emerging evidence of benefit from complement blockade with eculizumab, and no trial has tested anticoagulation specifically for biopsy-proven APSN [190,195,196]. Two strands of evidence give tentative direction to the non-immunosuppressive approach. Adding antiplatelet therapy to aPL-positive class III/IV patients without definite APS did not change the complete-response or relapse rate overall but was associated with an improvement in eGFR at three years confined to the lupus-anticoagulant-positive subgroup (*p* = 0.04), with no benefit in the lupus-anticoagulant-negative [197]. More strikingly, in patients with established APSN, early RAAS blockade—started within three months and continued at least twelve—reduced the risk of kidney-disease progression by roughly 90% (HR 0.11, 95% CI 0.02–0.59 for the 30%-eGFR-decline-or-ESKD endpoint) and improved eGFR independently of blood-pressure and proteinuria control [198].

## 9. Thrombotic Microangiopathy

The same asymmetry applies to thrombotic microangiopathy: the evidence concerns hard outcomes and acute kidney injury almost exclusively, with renal flare and remission seldom reported separately for patients with TMA, so that the soft-outcome side of this axis remains largely undocumented.

Renal TMA sits outside the ISN/RPS class yet repeatedly modifies its prognosis. Reported in anywhere from under 1% to about a quarter of biopsies depending on definition and population, it is frequently renal-limited—detectable on biopsy when systemic hematological criteria are absent—which makes histology the decisive diagnostic tool [5,199]. An Indian cohort sits at the upper end of that range, with TMA in 25.4% of biopsies [200]. Across cohorts the direction of effect is consistent: TMA marks a worse renal course. Its status as an independent driver, however, is genuinely contested, and that tension is the most interesting part of the story.

### 9.1. TMA and Hard Renal Outcome

Many cohorts identify TMA as an independent adverse predictor. In a Chinese series it carried a hazard ratio of 2.77 for the renal endpoint [40]; in a combined Chinese–Hong Kong cohort, five-year renal survival was 70% versus 95% with an adjusted hazard ratio of 7.16 [199]; in a Brazilian proliferative cohort, the composite renal endpoint reached 83% versus 33% with an independent hazard ratio of 2.68 [5]; in a propensity-matched cohort, TMA halved the probability of renal-function recovery (HR 0.50) and raised the risk of kidney replacement therapy roughly eleven-fold [41]; and in a matched cohort, renal survival was 68% versus 89% at three years (HR 4.81), the effect mediated by interstitial fibrosis and complement activation [201]. The vascular-spectrum cohort of Vidal-Montal and colleagues found TMA the single heaviest vascular predictor of ESKD (HR 12.99) [25]. A systematic review of 446 patients pooled these signals into a relative risk for renal outcome of about four at one year and 2.5 at five years, with five-year renal survival of only 56% [202]. Several focused series add concordant detail. In class IV LN complicated by TMA, the five-year renal survival was only 46.7% and patient survival 69.2%, with need for acute dialysis, treatment non-response and a high chronicity index identifying those reaching end-stage failure [203]. In the Indian cohort, TMA independently predicted a poorer treatment outcome (multivariate *p* < 0.001) and carried more treatment failure (38% versus 15.9%), more crescents and more tubular atrophy [200]. Most pointedly, lupus-associated TMA arising without proliferative LN behaved worse than class IV disease itself: 37.5% of such patients became dialysis-dependent versus none of the class IV controls, alongside deaths and a transplant [204].

A second group of studies tempers this. When outcomes were adjusted for baseline severity and chronicity, the apparent effect of TMA attenuated or disappeared: an end-stage hazard ratio of 3.77 fell to a non-significant 2.20 after adjustment [42], and in a cohort modeling the full spectrum of vascular lesions TMA was not independent once activity and chronicity scores were included, chronic TMA regaining significance only in the model from which the chronicity score was omitted [36]. A case–control study matched on disease severity found no significant outcome difference [205]. The reconciliation is that TMA is partly a marker of severe, chronic disease and partly an independent insult; the isolated, non-immune-complex form can even carry an excellent prognosis [206]. The microthrombotic lesion that underlies the spectrum follows the same logic. Glomerular thrombosis in LN was found mainly in diffuse proliferative disease and tracked with a markedly higher activity index (12.9 versus 5.4), trending toward—but not independently predicting—subsequent glomerular sclerosis and showing no significant association with anticardiolipin antibodies [207]. Immunostaining tells a parallel story: CD61-positive renal microthrombosis was present in 52% of proliferative biopsies and more prevalent with aPL positivity (OR 4.4, 95% CI 1.5–12.8), yet neither microthrombi nor C4d correlated independently with long-term renal function, macrophage infiltration proving the better marker of clinical activity [208]. Even the antibody link to TMA outcome is inconsistent, anticardiolipin positivity not being significantly associated with prognosis in the class IV TMA series [203]. Stratification of aPL-positive patients by renal histology helps order this heterogeneity: hierarchical clustering identified a TMA cluster with the worst twelve-month renal response (complete response 30.4%, no response 47.8%) linked to thrombotic events, triple positivity and a high aGAPSS, a hyperplastic-vasculopathy cluster with intermediate outcome, and a subendothelial-oedema cluster—confined to SLE/LN patients—with the best response [209].

### 9.2. Treatment Signals and the Complement Question

Because mechanism bears on therapy, the treatment data matter even though they are weak. Plasma exchange was used preferentially in TMA but showed no demonstrable renal benefit in matched comparisons, with double-filtration plasmapheresis helping mainly the subset requiring acute dialysis [42,199,202]. A more recent randomized comparison gives a slightly more favorable read: in 100 patients with TMA-LN, both plasma exchange and cyclophosphamide induction (with steroids) achieved significant renal, immunological and hematological improvement over twelve months, with plasma exchange yielding faster early renal response (78% versus 30% at three months) and a more stable long-term creatinine, while no baseline factor predicted response in either arm [210]. The recurrent mechanistic thread is complementary, and complement inhibition is recommended for the complement-mediated, atypical-HUS-like phenotype on anecdotal rather than pooled evidence [196,202]. TMA is therefore best framed as a prognostically important, biopsy-defined modifier whose independent weight depends on how much baseline chronicity is already in the model and whose therapy remains to be defined by trials.

## 10. Repeat and Per-Protocol Biopsy and Renal Flare

The biopsy is usually treated as a baseline event, but its prognostic value increases when it is repeated. Two messages emerge: histology at a second biopsy predicts hard outcomes better than the first; and residual histological activity at a per-protocol biopsy predicts flare and governs the safety of treatment withdrawal—both because clinical and serological markers track histology poorly, a dissociation seen in roughly a quarter to nearly half of clinical responders [211].

### 10.1. The Second Biopsy Predicts Hard Outcome

Where the first biopsy often fails to stratify long-term function, the second succeeds. In a large multicenter Italian cohort, neither the activity index, the chronicity index nor proteinuria at first biopsy predicted ESKD, whereas all three did at the second biopsy (activity index HR 1.20; chronicity index HR 1.41), with ESKD concentrated in patients re-biopsied for a nephritic flare [43]. The single most striking quantitative endorsement comes from a 141-patient repeat-biopsy cohort: histological worsening on the second biopsy quadrupled the risk of ESKD (HR 4.2) and death (HR 4.3), and a short interval between biopsies (<1 year, signaling refractory disease) raised these risks more than ten-fold (ESKD HR 13.7; death HR 16.9) [45]. Earlier serial-biopsy data agree: extensive crescents and a chronicity index of at least 5 at repeat biopsy predicted creatinine doubling when baseline clinical features did not [46], a rising chronicity index at a per-protocol biopsy predicted sustained functional loss [47], and the chronicity index at a post-induction biopsy—not the activity index or the clinical response category—predicted long-term chronic kidney disease [212,213]. The mechanistic study behind these observations showed that it is the response of inflammation to therapy at a six-month biopsy, not the static immune-deposit burden, that determines whether a relapse progresses to a hard outcome and that interstitial fibrosis can regress with an attendant excellent prognosis [135,214].

A consistent body of repeat-biopsy work confirms that it is the histology read at the second sampling—particularly the chronicity it captures—that carries the prognostic weight [48,215,216,217,218]. In a paired first-and-second biopsy series, the activity index at the second biopsy predicted creatinine doubling (relative risk 1.4 for an index of 1–2 and 1.68 for an index above 2), whereas the baseline activity index did not (*p* = 0.688), and a second-biopsy chronicity index of at least 7 conferred a relative ratio of 2.18, with ten-year renal survival falling from 93% in complete responders to 41% in non-responders [48]. In a large international cohort, the ISN/RPS class at repeat biopsy stratified follow-up creatinine far better than the first-biopsy class (mean follow-up creatinine 2.8 versus 0.8 mg/dL for IV-G versus IV-S at repeat biopsy), and class II at repeat biopsy—unlike class II at presentation—was a genuinely favorable indicator [215]. In a smaller clinically indicated series, second-biopsy chronic lesions (tubular atrophy 90 versus 38%, interstitial fibrosis 50 versus 13%, vascular lesions 60 versus 19%) together with absence of complete remission at twelve months predicted CKD progression, which occurred in 38% [216]; and in a withdrawal-oriented cohort, the chronicity index was the dominant correlate of ESKD/CKD on bivariate analysis, alongside older age at diagnosis [217]. Repeat biopsy performed for nephrotic syndrome or AKI after immunosuppression likewise showed that a chronicity index of at least 5, a serum creatinine of at least 140 µmol/L and AKI at the second biopsy—but not AKI at the first—independently predicted the composite of creatinine doubling, ESKD or death. The cumulative penalty of relapse itself reinforces this picture: in a 441-patient flare cohort each successive renal flare independently lowered the chance of complete response and worsened kidney and patient survival, even after adjustment for chronic damage (third flare versus first: doubling of creatinine-adjusted HR 1.61, 30% eGFR decline HR 1.69, death HR 3.85), with the histologic chronicity index again an independent predictor of every long-term outcome [218].

The dynamics of how activity resolves and chronicity accrues clarify why the second biopsy is informative. Serial sampling shows that histological activity clears slowly and biphasically—hyperinflammatory crescents and necrosis regress early, while endocapillary hypercellularity, subendothelial hyaline deposits and interstitial inflammation persist—whereas chronic damage accumulates rapidly and early, irrespective of whether histological remission is reached [219]; in the centrally-read MAINTAIN substudy, the activity index fell and the chronicity index rose modestly over two years, with no difference between azathioprine and mycophenolate maintenance, and persistently active histology at re-biopsy was not associated with a worse functional outcome [220]. Glomerular injury at the index biopsy appears to seed this tubulointerstitial scarring: proliferative disease and crescents at the index biopsy predicted progression of interstitial fibrosis and tubular atrophy from none–mild to moderate–severe on clinically indicated re-biopsy (57% versus 17%, *p* = 0.002), affecting roughly half of patients [221], and chronicity markers such as IF/TA above 30% and thrombotic microangiopathy at the second biopsy independently predicted a poorer treatment response [222].

Finally, the histological class itself is unstable: it switches between proliferative and non-proliferative patterns in roughly half of repeat biopsies, so that pure membranous disease preceded by proliferative nephritis is not benign and carries a real risk of ESKD [223,224]. Multiple independent series quantify and qualify this instability [37,216,225,226,227,228]. Class transition is reported in 40–75% of repeat biopsies and is not predictable from baseline clinical, biochemical or pathological variables (75% transformation in one 156-patient flare series, with only lower baseline creatinine and a longer inter-biopsy interval associated with change) [226,228]. The risk is directionally asymmetric: proliferative lesions rarely revert to pure non-proliferative disease (7.7% in one series), whereas non-proliferative lesions frequently progress to proliferative forms (63.2%), so a low threshold for re-biopsy is best reserved for patients whose reference biopsy was non-proliferative [225,227,228]. Across these cohorts, re-biopsy modified immunosuppression in a substantial minority to a majority of cases (31% in one series, with every patient who switched from non-proliferative to proliferative escalating therapy), and the second biopsy consistently carried higher chronicity, crescent burden and creatinine than the first [37,227,228].

### 10.2. Per-Protocol Biopsy, Flare Prediction and Safe Withdrawal

The clearest practical use of repeat histology is to read residual activity in patients in apparent clinical remission. Across per-protocol cohorts, roughly a quarter to nearly half of patients in apparent clinical remission harbor residual histological activity, and that activity predicts flare [213,229,230]. In an Argentine withdrawal study, an activity index of at least 1 at the second biopsy conferred an odds ratio of 31.7 for flare after stopping immunosuppression [229]; a Belgian per-protocol cohort found the activity index to predict relapse independent of proteinuria [47]; residual active lesions at a protocol biopsy in clinical remission predicted flare with an odds ratio above 20 in one cohort [49]; and an indicated-biopsy series found flare in 78% of patients with persistent proliferative lesions versus 35% without [231]. Conversely, histological remission identifies patients who can stop treatment safely: an activity-index-guided protocol withdrew maintenance immunosuppression in the 72% with an activity index of 0 and recorded a flare rate of only 9% with no ESKD [232], and longer sustained clinical remission predicts histological remission [233].

These conclusions are reinforced by further withdrawal and surveillance cohorts [211,217,219,234,235]. In an activity-index-guided treatment regimen, only 8 of 74 patients (7.3%) who stopped immunosuppression with an activity index of 0 subsequently flared, and persistent residual immune deposits at the final biopsy were far more common when the activity index remained at least 1 (IgG 94% versus 55%) [219]. In a separate withdrawal cohort, stopping immunosuppression precipitated a flare in about 23% of patients in stable remission, with antimalarial maintenance, older age at discontinuation and remission lasting more than three years before discontinuation each independently protective against flare [234]; the protective effect of antimalarials against renal flare was echoed in a per-protocol series in which absence of antimalarial therapy was the strongest predictor of flare on multivariate analysis (OR 21.7) [217]. In severe pediatric new-onset disease, a six-month follow-up biopsy documented histological improvement in 12 out of 13 patients and guided the transition from cyclophosphamide to mycophenolate, while the flares that did occur within twelve months were serological and attributable to non-compliance [235].

Where biopsy is not repeated, the limits of clinical and serological surveillance become the recurring theme. In two Korean complete-responder cohorts, renal relapse was frequent (38% at 5 years and up to 68% at 20 years in one series of 296 patients) yet was predicted by demographic and serological features—young-age onset of lupus (HR 0.779 per decade), anti-Ro seronegativity and persistent anti-dsDNA positivity at six months—rather than by any pathological finding, including class, activity index, chronicity index or crescents [236,237]. Consistent with this dissociation, the dynamic first-year course of surveillance laboratory parameters (proteinuria, albumin, anti-dsDNA, C3, and C4) did not differ in the number of flares a patient eventually sustained [218].

The same clinical–histological correlates of outcome extend beyond the flare setting and reinforce why protocolized re-assessment is warranted. The severity of acute kidney injury at presentation, graded by KDIGO stage, was independently associated with progression to ESKD in diffuse proliferative nephritis (adjusted HR 3.8; ten-year ESKD-free survival falling from 95% with no AKI to 15% with AKI-3) alongside great crescents above 30%, anemia and heavy proteinuria [51]. Failure to attain or sustain remission likewise drove long-term function: in a 322-patient cohort, CKD developed in 22% and was predicted by relapse (HR 2.81) and resistance to induction (HR 8.12), rather than by histological class or proteinuria severity [53]. Persistent microscopic hematuria—present in about 37% of patients during follow-up—independently predicted the composite of death, ESKD or creatinine doubling (time-dependent adjusted HR 3.40, rising to HR 9.32 in the biopsy-stratified subgroup), supporting hematuria as a remission marker beyond proteinuria [52]. Finally, the pattern of complement activation refines histological risk: among hypocomplementemic patients, classical-pathway activation (low C3 and C4) accompanied a higher renal activity index and more class IV disease and was associated with a significantly poorer composite renal outcome than alternative-pathway activation (Kaplan–Meier *p* = 0.037) [238].

## 11. Key Messages, Synthesis and Future Directions

### 11.1. Key Messages

KM1. Chronicity, rather than activity, is the histological dimension most consistently associated with hard renal outcomes: the chronicity index and its components (glomerulosclerosis, fibrous crescents, and interstitial fibrosis/tubular atrophy) predict ESKD across cohorts and classifications. Whether they do so independently of the baseline eGFR and proteinuria already available to the clinician is a separate question, and one this literature still answers inconsistently.

KM2. The activity index is a weak predictor of hard outcomes overall, but two of its components—cellular/fibrocellular crescents and fibrinoid necrosis—can predict them independently, and activity that persists at a repeat biopsy regains prognostic weight.

KM3. The tubulointerstitium is at least as important as the glomerulus for hard outcomes and has historically been under-scored.

KM4. The ISN/RPS IV-S vs. IV-G subdivision does not predict outcome; the 2018 revision, by incorporating the activity and chronicity indices, predicts prognosis better than the 2003 system. Class IV nonetheless remains a robust univariate and, in several cohorts, independent predictor—its independence conditional on the model.

KM5. Several entities outside the classification modify prognosis: lupus podocytopathy (high relapse, FSGS-subtype risk), vascular immune deposits and lupus vasculopathy (not benign), APSN, and TMA (adverse, its independence attenuating after adjustment for chronicity).

KM6. Immune-deposit intensity and a full-house pattern are largely diagnostic, not prognostic; but deposit location (tubular basement membrane, adverse) and type (IgA and exostosin, favorable) carry information.

KM7. Repeat and per-protocol biopsy add prognostic information beyond the baseline: the second biopsy predicts ESKD where the first does not, and a short inter-biopsy interval signals a more than ten-fold excess risk.

### 11.2. Synthesis

**Figure 2:**
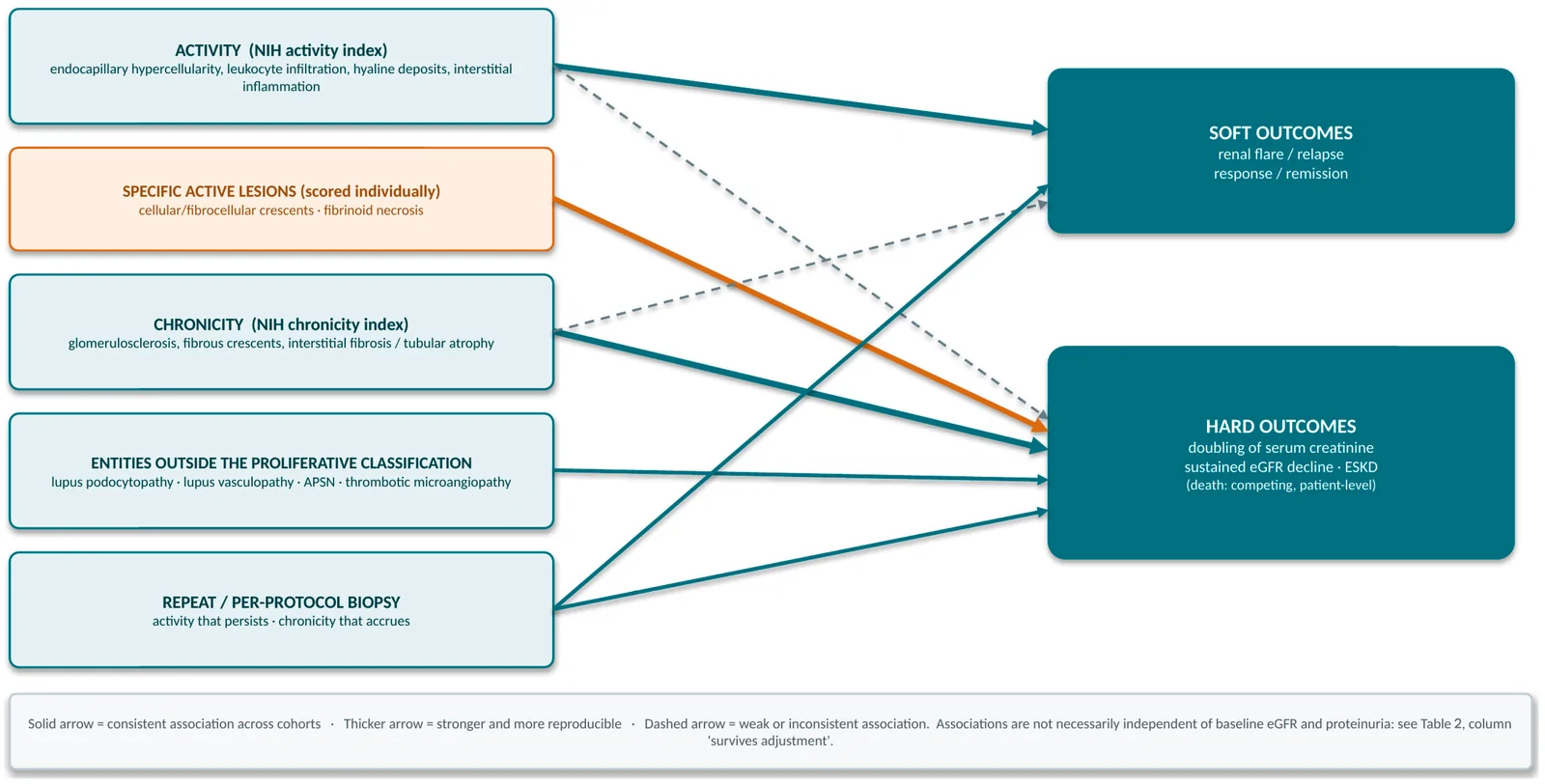
How each histological axis maps onto soft and hard renal outcomes in lupus nephritis. Solid arrows denote associations that are consistent across cohorts and thicker arrows those that are stronger and more reproducible; dashed arrows denote weak or inconsistent associations. Associations shown are not necessarily independent of baseline eGFR and proteinuria; Table 2 records, for each axis, whether the association survived multivariable adjustment.

Across all eight axes, a single organizing principle emerges, summarized axis by axis in Table 2 and schematically in Figure 2: chronicity predicts hard outcomes, activity predicts response and flare. The chronicity index and its constituent lesions—glomerulosclerosis; fibrous crescents; and, above all, tubulointerstitial fibrosis and atrophy—are the most consistent predictors of ESKD across cohorts and successive classifications, whereas the activity index, as a composite, is weak. The principal qualification is that two active lesions, cellular/fibrocellular crescents and fibrinoid necrosis, behave as exceptions and can independently signal hard outcomes; and activity reclaims prognostic weight when it is extreme or when it persists at a repeat biopsy. It is also possible that the prognostic value of the activity index has genuinely eroded over the decades, as progressively more effective immunosuppression has weakened the link between active lesions and progression—a possibility the heterogeneous historical cohorts reviewed here cannot resolve.

Two further messages cut across the axes. The tubulointerstitium is at least as important as the glomerulus for hard outcomes and has been historically under-scored—a gap the 2018 revision began to close. And several prognostically important entities lie outside the proliferative classification: lupus podocytopathy, vascular immune-complex deposits and lupus vasculopathy, APSN, and TMA. By contrast, the immune-deposit burden that defines the class carries little independent prognostic information, with the instructive exceptions of tubular-basement-membrane deposits and of IgA and exostosin positivity. Finally, repeat biopsy adds value beyond the baseline: the second biopsy predicts ESKD where the first does not, and residual histological activity at a per-protocol biopsy predicts flare.

### 11.3. Two Caveats: Circularity and the Competing Risk of Death

Two caveats qualify this organizing principle, and the evidence assembled here does not fully resolve either. The first concerns circularity. The chronicity index is built from glomerulosclerosis, fibrous crescents and interstitial fibrosis with tubular atrophy—that is, from damage that has already occurred—so that a kidney bearing more irreversible scarring should fare worse is close to a tautology. The clinically useful question is not whether chronicity predicts, but whether it adds to the baseline eGFR and proteinuria the clinician already has, and on that narrower question the evidence is genuinely mixed: chronicity retained independent weight after adjustment for kidney function in several large cohorts [13,26,29]; the histological class lost independence in others once function, proteinuria and chronicity were entered [71,72]; and the foundational demonstration that the biopsy adds information beyond clinical data rested on glomerular scarring rather than on class [140]. We have therefore tried throughout to distinguish predicting an outcome from predicting it independently of baseline function, because most of the disagreement in this literature turns on precisely that distinction. The second caveat concerns death. We counted death among the hard outcomes, following the convention of the source studies, but mortality in LN is driven substantially by infection and cardiovascular disease rather than by progression of the renal lesion; where a composite of ESKD or death is reported, part of the endpoint is only loosely coupled to the histology being scored. Death is thus better regarded as a competing, patient-level outcome than as a renal one, and wherever the source papers allowed it we have given the renal endpoint separately.

### 11.4. Limitations

This is a narrative synthesis and carries the limitations of that design. Records were screened by a single reviewer using relevance-based judgement, without duplicate screening or a formal adjudication procedure; no risk-of-bias instrument was applied and no quantitative synthesis was attempted, so Figure 1 should be read as a record of how the literature was assembled rather than as evidence of systematic method. Fifty studies meeting the eligibility criteria are not cited because their full text could not be obtained through institutional subscriptions or open-access repositories; interlibrary loan and author contact were not systematically pursued. This is nearly a fifth of the eligible literature and is unlikely to be a random fifth, since older work, smaller journals and less well indexed series are systematically harder to retrieve and may differ both in their findings and in their reporting quality; the exclusion of non-English-language studies compounds the same bias. The literature itself imposes further limits: outcome definitions are heterogeneous, the scoring system changed twice over the period covered, inter-observer reproducibility of the indices is only moderate, and the therapeutic era conditions every estimate. Finally, effect estimates from different cohorts appear near one another for narrative economy; they are not pooled, are not adjusted for the same covariates and are not directly comparable, and Table 1 is provided so that the outcome definition and adjustment status behind each figure remain visible.

## 12. Conclusions

Several gaps follow. Outcome definitions remain heterogeneous, and LN research has not adopted the validated eGFR-decline and eGFR-slope surrogates now standard elsewhere in nephrology, limiting comparison across cohorts and eras [7,9]. The reproducibility of activity and chronicity scoring is only moderate; the independence of the histological class versus chronicity and renal function differs across cohorts and deserves harmonized, adequately powered multivariable analysis; and prospective, multi-ethnic data are scarce precisely for the entities outside the classification—podocytopathy, the vascular lesions, APSN and TMA—for which treatment remains to be defined by trials now under way.

The tools that will reshape this field are already visible. Molecular and transcriptomic profiling of biopsy tissue distinguishes treatment responders from non-responders in ways that conventional scoring does not, and intrarenal molecular signatures may prove more reproducible than morphology; glomerular mTORC1 activation has been linked to crescentic burden and to independent prognostic weight, suggesting that pathway-level readouts may refine the activity axis. Digital pathology and artificial-intelligence-assisted scoring address the reproducibility ceiling directly by making lesion quantification continuous and observer-independent rather than semiquantitative and consensual. If these approaches deliver, the prognostic question will shift from how well a pathologist can grade chronicity to which molecular programs remain active in a kidney that already looks scarred.

A pervasive and under-acknowledged confounder runs through the entire literature: the therapeutic era. The prognostic weight of any histological lesion is conditioned by the treatment patients received, and treatment has changed radically across the four decades these cohorts span—from the early corticosteroid and intravenous-cyclophosphamide era of the NIH and Lupus Nephritis Collaborative Study cohorts [82,90,135] through the mycophenolate-and-cyclophosphamide era of most modern series to the contemporary multitarget era of belimumab, voclosporin and obinutuzumab. There is direct histological evidence that treatment modifies the very lesions we score: cyclophosphamide slowed the accrual of chronic damage on per-protocol repeat biopsy relative to azathioprine [239], antimalarials independently reduced renal damage [240], and maintenance immunosuppression reduced nephritic flares [50]—yet across a multicenter cohort accrued over more than three decades, hard renal and patient outcomes did not measurably improve [17]. A lesion that predicted progression when the only option was cyclophosphamide may predict far less once effective, better-tolerated regimens are available—the most plausible explanation for the apparent waning of the activity index. Compounding this, cohorts differ in whether their analyses are adjusted for treatment at all: some adjust for induction regimen or response, many do not, and almost none account for the therapy actually available in their era. The result is a genuine knowledge gap—for most lesions we do not know whether their prognostic significance survives adjustment for treatment and for era-specific therapeutic options. Studies relating histology to outcome should therefore report the treatment era explicitly and model treatment as a covariate so that the prognostic value attributable to the biopsy can be separated from that attributable to what was done after it. The practical message for the clinician, however, is already clear: the prognostic content of the LN biopsy lies less in its class label than in its chronicity, its tubulointerstitium, and the entities the classification does not name—and, in difficult cases, in reading it again.

## Figures and Tables

**Table 1:** Representative cohorts underlying each axis, with population, outcome definition as used in the source study, effect estimate and adjustment status. Estimates were taken from the full text of each study. “NR” indicates that the item was not reported or could not be established from the source. Estimates from different rows are not directly comparable.

**Classification**						
**Study [Ref.]**	**N**	**Population**	**Outcome (As Defined)**	**Histological Predictor**	**Effect Estimate**	**Adjustment**
Yokoyama 2004 [10]	60	Japanese, biopsies 1973–2000, reclassified per ISN/RPS 2003	ESRF	ISN/RPS class IV (-S or -G)	HR 14.82 (95% CI 1.42–155.3), *p* = 0.025; stepwise model HR 25.55, *p* = 0.0021; WHO 1995 class IV HR 2.16 (95% CI 0.15–31.9), *p* = 0.57	Multivariable (Cox)
Tang 2015 [11]	681	Adults, West China Hospital, biopsies 2006–2012	ESRD	Class IV or VI	HR 7.17 (95% CI 3.10–16.56), *p* < 0.001	Multivariable
Norby 2017 [12]	178	Norwegian, 94% Caucasian, registry 1988–2007	All-cause mortality	WHO class IV	HR 2.6 (95% CI 1.2–5.7), *p* = 0.017	Multivariable (age, creatinine, hypertension)
Shao 2019 [13]	1945	Chinese, Nanjing registry 1994–2010	ESRD	Chronicity index; activity index	CI adj HR 1.31 (95% CI 1.23–1.39), *p* < 0.001; AI adj HR 1.05 (95% CI 1.01–1.09), *p* = 0.04	Multivariable
Moroni 2018 [14]	499	Four Italian centers 1970–2016, 90% Caucasian	ESKD	Chronicity index; activity index	CI RR 1.29 (95% CI 1.11–1.49), *p* < 0.001; AI RR 1.10 (95% CI 1.02–1.19), *p* = 0.02	Multivariable
Mok 1999 [15]	183	Southern Chinese, Hong Kong 1976–1997	ESRF	WHO class IV	Multivariable Cox β 1.55 (SE 0.68), *p* = 0.02; univariable OR 24.6 (95% CI 3.3–187)	Multivariable
Haring 2012 [16]	8 pooled studies	Class IV LN, IV-S vs. IV-G	Doubling of creatinine or ESRD	Class IV-G vs. IV-S	RR 1.08 (95% CI 0.68–1.70), not significant	Meta-analysis
Faurschou 2010 [17]	100	Danish multicenter 1971–1995, 98% white	Death	WHO class III; class VI	Class III HR 8.0 (95% CI 3.1–20.6); class VI HR 38.9 (95% CI 7.1–212.7)	NR
**Individual Lesions**						
**Study [Ref.]**	**N**	**Population**	**Outcome (As Defined)**	**Histological Predictor**	**Effect Estimate**	**Adjustment**
Rijnink 2017 [18]	105	Leiden 1987–2011; 65% white	ESRD	Fibrinoid necrosis; fibrous crescents; IF/TA ≥ 25%	Necrosis HR 1.08 per % (95% CI 1.02–1.13); fibrous crescents HR 1.09 per % (95% CI 1.02–1.17); IF/TA HR 3.89 (95% CI 1.25–12.14)	NR
Zhang 2016 [19]	788	Sun Yat-sen University 1996–2011	Doubling of creatinine + ESRD	Proportion of crescents; presence of crescents	Proportion HR 1.16 per 10% (95% CI 1.00–1.34), *p* = 0.049; presence not significant (HR 0.82, *p* = 0.61)	Multivariable (age, sex, eGFR, proteinuria, AI, CI)
Cai 2018 [20]	231	Class III/IV (±V), Zhejiang 2004–2015	Doubling of creatinine or ESRD	Cellular crescents; fibrocellular crescents; severe tubular atrophy	1.040 (95% CI 1.015–1.066); 1.085 (95% CI 1.013–1.163); 5.348 (95% CI 1.278–22.373)	NR
Yu 2010 [21]	313	Five centers, North China 2003–2007	Doubling of creatinine or ESRD	Interstitial inflammation; tubular atrophy; interstitial fibrosis	1.847 (95% CI 1.229–2.777); 2.350 (95% CI 1.019–2.932); 1.956 (95% CI 1.237–2.235)	Multivariable (stepwise Cox)
Leatherwood 2019 [22]	202	Brigham and Women’s 1990–2017; 35% African American	ESRD; death	Moderate/severe IF/TA	ESRD HR 5.18 (95% CI 2.53–10.59); death HR 4.19 (95% CI 1.27–13.81)	Multivariable (age, sex, race, creatinine, era, class)
Liao 2023 [23]	526	Taiwanese, 2006–2019	Kidney failure	Tubular atrophy; tubulointerstitial inflammation	Atrophy HR 2.28 (95% CI 1.66–3.14); TII HR 3.13 (95% CI 1.34–7.33)	Multivariable
Wilson 2018 [24]	301	Yale New Haven 1998–2014; multi-ethnic	ESRD/RRT	Interstitial inflammation; IF/TA	Multivariable: inflammation HR 1.58 (95% CI 1.06–2.36); IF/TA HR 2.20 (95% CI 1.29–3.74). Univariable gradient IF/TA >50% vs. <5% HR 13.99 (95% CI 4.91–39.83)	Multivariable
Vidal-Montal 2026 [25]	135 patients (195 biopsies)	Spanish, ISN/RPS 2018 with modified NIH indices	ESRD	Moderate/severe IF/TA; TMA	IF/TA HR 8.93 (95% CI 2.27–34.48); TMA HR 12.99 (95% CI 2.12–76.92); NIH-TII not independent (*p* = 0.68)	Multivariable
**Activity and Chronicity Indices**						
**Study [Ref.]**	**N**	**Population**	**Outcome (As Defined)**	**Histological Predictor**	**Effect Estimate**	**Adjustment**
Cuéllar-Gutiérrez 2026 [26]	307	Mayo Clinic 1992–2023; 63.5% White; median follow-up 11 yr	ESKD	NIH chronicity index (per point); IF/TA	CI HR 1.53 (95% CI 1.38–1.68); fully adjusted 1.40 (95% CI 1.26–1.56); IF/TA HR 3.70 (95% CI 2.40–5.70); neither index predicted mortality (adj HR 1.05, 95% CI 0.91–1.22)	Multivariable
Tinajero-Sánchez 2025 [27]	430	Mexican, proliferative LN 2008–2020; median follow-up 97 months	ESKD	Chronicity index; activity index	CI HR 1.45 (95% CI 1.32–1.61); AI HR 1.05 (95% CI 0.99–1.11), not significant; severe fibrinoid necrosis HR 5.87 (95% CI 1.42–24.3)	NR
Zhang 2026 [28]	172	Prospective multicenter (AMP RA/SLE), centrally scored	Sustained ≥40% eGFR decline or ESKD	Chronicity index; activity index	CI HR 1.33 (95% CI 1.18–1.50), *p* < 0.001; CI ≥7 HR 3.52 (95% CI 1.04–11.91); AI and ISN class not predictive	NR
Zavala-Miranda 2023 [29]	440	Mexican mestizo, 2008–2018; median follow-up 79 months	ESKD	Chronicity index	Adjusted HR 1.13 (95% CI 1.03–1.25), *p* = 0.009	Multivariable
Peng 2018 [30]	680 (447 biopsied)	West China Hospital 2010–2016	Doubling of creatinine/GFR < 15/RRT/death	Chronicity index; cellular crescents	CI HR 1.348 (95% CI 1.214–1.498); cellular crescents HR 1.440 (95% CI 1.223–1.695)	NR
Contreras 2005 [31]	213	Miami; 47% Hispanic, 44% African American	Doubling of creatinine/ESRD/death	Chronicity index (per point)	HR 1.18 (95% CI 1.07–1.30), *p* = 0.001; activity index significant on univariable analysis only	Multivariable
Singh 2011 [32]	163	UT Southwestern 1999–2008; predominantly African American and Hispanic	ESRD or death	Chronicity index; activity index	CI OR 1.771 (95% CI 1.273–2.464), *p* < 0.001; AI not significant	Multivariable
Nakagawa 2021 [33]	66	Single Japanese center 1977–2018; modified NIH indices	ESKD or death	Modified chronicity index (moderate; high)	Moderate HR 6.17 (95% CI 1.14–33.20); high HR 20.20 (95% CI 1.13–359.82); activity index not associated	Multivariable (age, creatinine)
**Vascular Lesions, APSN and Thrombotic Microangiopathy**						
**Study [Ref.]**	**N**	**Population**	**Outcome (As Defined)**	**Histological Predictor**	**Effect Estimate**	**Adjustment**
Banfi 1991 [34]	285	GISNEL, 20 Italian centers, 1960–1988	ESKD	Any renal vascular lesion	HR 5.49 (95% CI 2.6–11.3), *p* < 0.001	Univariable
Ma 2025 [35]	225	Chinese, 2008–2021	Death/ESRD/≥30% creatinine rise	Any renal vascular lesion	Adjusted HR 1.920 (95% CI 1.011–3.645), *p* = 0.046; unadjusted 2.123 (95% CI 1.176–3.823)	Multivariable
Mejía-Vilet 2017 [36]	429	Hispanic, Mexico City 2008–2015	ESRD	True renal vasculitis; arterial sclerosis; chronic TMA	Vasculitis HR 2.65–2.98; arterial sclerosis HR 1.90 (95% CI 1.21–2.97); chronic TMA HR 2.14 (95% CI 1.06–4.32)	Multivariable; no vascular lesion independent of activity/chronicity scores
Lin 2025 [37]	84 patients (107 biopsies)	Chinese children, Hong Kong 2004–2020	Advanced CKD/kidney failure/death	Non-inflammatory necrotizing vasculopathy	NNV HR 7.08 (95% CI 1.67–30.03); any vascular lesion HR 3.45 (95% CI 0.86–13.80), NS	NR
Moroni 2004 [38]	111	Italian, prospectively tested for aPL	Chronic renal insufficiency	Chronicity index (aPL as co-predictor)	CI RR 1.34 (95% CI 1.16–1.54); aPL positivity RR 2.2 (95% CI 1.06–4.48)	Multivariable
Gerhardsson 2015 [39]	112	Karolinska cohort 1995–2011	ESRD	Histological APS nephropathy vs. pure LN	OR 5.8 (95% CI 1.7–19.7), *p* = 0.008	NR
Strufaldi 2021 [5]	253	Brazilian, proliferative LN 2012–2018	eGFR < 15 or dialysis or death	Renal TMA	HR 2.68 (95% CI 1.42–5.03), *p* = 0.002	Multivariable
Song 2013 [40]	148	Peking University First Hospital 2002–2008	ESRD or doubling of creatinine	Renal TMA	HR 2.772 (95% CI 1.009–7.617), *p* = 0.048	Multivariable
Zhang 2025 [41]	1218	Sun Yat-sen, 2001–2023; propensity-score-matched	Kidney replacement therapy	Renal TMA	Multivariable HR 6.37 (95% CI 3.28–12.40); matched HR 11.20 (95% CI 2.50–50.00)	Multivariable + PSM
Massicotte-Azarniouch 2022 [42]	17 cases, matched controls	North Carolina, predominantly Black, 1980–2020	ESKD	Renal TMA	Unadjusted HR 3.77 (95% CI 1.24–11.41); adjusted for creatinine and proteinuria 2.20 (95% CI 0.63–7.71), NS	Both reported
**Repeat and Per-Protocol Biopsy**						
**Study [Ref.]**	**N**	**Population**	**Outcome (As Defined)**	**Histological Predictor**	**Effect Estimate**	**Adjustment**
Gatto 2022 [43]	92	Four Italian centers 1990–2018; mean follow-up 17.9 yr	ESKD	Chronicity and activity indices at the SECOND biopsy	CI HR 1.41 (95% CI 1.09–1.82), *p* = 0.008; AI HR 1.20 (95% CI 1.03–1.41), *p* = 0.022; first-biopsy indices not predictive	Multivariable
Moroni 2022 [44]	61 (of 203)	Italian, second biopsy for flare or per protocol	≥30% fall in creatinine clearance/eGFR	Chronicity index > 4; activity index > 3 at second biopsy	CI OR 2.905 (95% CI 1.285–6.566); AI OR 3.230 (95% CI 1.275–8.183)	Multivariable
Arriens 2017 [45]	141	Dallas 1999–2015	ESKD; death	Biopsy worsening at second biopsy	ESKD HR 4.2, *p* < 0.001; death HR 4.3, *p* = 0.022; interval <1 yr, ESKD HR 13.7	Multivariable
Moroni 1999 [46]	31 patients (38 repeat biopsies)	Italian, median follow-up 10.5 yr	Doubling of plasma creatinine	Chronicity index ≥ 5; cellular crescents > 30% at repeat biopsy	CI ≥ 5 RR 23.6 (95% CI 5.1–110); crescents RR 7.2 (95% CI 2.2–23.5)	Multivariable
Parodis 2020 [47]	42	Per-protocol repeat biopsy (Euro-Lupus/MAINTAIN), median 24 months	Sustained creatinine ≥120% of baseline	Chronicity index at repeat biopsy	CI > 3 HR 12.4 (95% CI 1.4–106.7); continuous CI HR 1.8 (95% CI 1.1–2.9); baseline indices not associated	NR
Alsuwaida 2012 [48]	77 patients (144 biopsies)	Riyadh 1996–2009; median follow-up 8.7 yr	Doubling of serum creatinine	Activity and chronicity indices at second biopsy	AI > 2 RR 1.68 (95% CI 1.3–2.2); CI ≥ 7 RR 2.18 (95% CI 1.41–3.37); baseline indices not predictive	NR
Hou 2024 [49]	114	Two Chinese centers; per-protocol biopsy at median 7.3 months	Renal flare within 3 years	Activity index > 1 at second biopsy	OR 23.1 (95% CI 5.1–103.8), *p* < 0.001	Univariable; multivariable model retained anti-dsDNA and crescents
Mok 2004 [50]	189	Chinese, class IV, five Hong Kong hospitals	Doubling of serum creatinine	Nephritic flare; chronicity score	Nephritic flare HR 17.7 (95% CI 1.59–198), *p* = 0.02; chronicity score HR 1.53 (95% CI 1.14–2.07) univariable only	Multivariable for flare
**Clinical–Histological Interfaces**						
**Study [Ref.]**	**N**	**Population**	**Outcome (As Defined)**	**Histological Predictor**	**Effect Estimate**	**Adjustment**
Chen 2020 [51]	167	Chinese, diffuse proliferative LN with AKI, 2000–2014	ESRD; ESRD/death	Crescents > 30% (KDIGO AKI stage as co-predictor)	Crescents > 30%, ESRD HR 2.1 (95% CI 1.2–3.6); AKI stage, ESRD/death adjusted HR 3.8 (95% CI 2.1–6.7)	Multivariable
Chen 2026 [52]	178 (103 biopsied)	Chinese, newly diagnosed LN 2016–2022	Death/ESRD/doubling of creatinine	Persistent hematuria (biopsy subgroup stratified by pathology)	Adjusted HR 3.40 (95% CI 1.06–10.90); biopsy subgroup 9.32 (95% CI 1.05–82.65)	Multivariable
Moon 2011 [53]	322	Korean, 1985–2010	CKD (eGFR < 60 for ≥3 months)	Clinical predictors (histology not independent)	Delayed-onset LN HR 2.904 (95% CI 1.430–5.894); eGFR <60 at onset HR 7.458 (95% CI 3.823–14.546)	Multivariable

**Table 2:** Synthesis of the eight histological axes against soft and hard renal outcomes. Representative estimates are single, verified examples chosen for cohort size or design; they are not pooled and are not directly comparable across rows because outcome definitions, populations, treatment eras and adjustment sets differ (see Table 1). “Survives adjustment” refers to whether the histological variable retained significance in a multivariable model that included baseline kidney function and/or chronicity.

Histological Axis	Soft Outcomes (Flare, Response/Remission)	Hard Outcomes (Creatinine Doubling, Sustained eGFR Loss, ESKD, Death)	Representative Estimate (Cohort)	Survives Adjustment?
ISN/RPS class and its 2018 revision (Section 3)	Weakly related; relapse is governed mainly by depth of remission and maintenance strategy	Proliferative classes carry the worst kidney survival	Class IV or VI, ESRD: HR 7.17 (95% CI 3.10–16.56), n = 681 [11]	Inconsistent—independent in several cohorts [11,15], lost once kidney function, proteinuria and chronicity are entered [71,72]
Individual glomerular lesions (Section 4.1)	Cellular crescents adversely predict remission	Cellular/fibrocellular crescents and fibrinoid necrosis independently signal hard outcomes	Cellular crescents, doubling of creatinine or ESRD: HR 1.040 per 1% (95% CI 1.015–1.066), n = 231 [120]	Yes, in several cohorts [20,118,120]
Tubulointerstitial compartment (Section 4.2)	Limited data	Strongest and most reproducible single-lesion signal	Moderate/severe IF/TA, ESRD: HR 5.18 (95% CI 2.53–10.59), n = 202 [22]	Yes—independent of ISN/RPS class [22,23,132]
Immune deposits (Section 4.3)	Largely diagnostic rather than prognostic	Deposit burden non-predictive; location and antigen carry information	Exostosin positivity in membranous LN: milder phenotype, better renal survival [146]	Not for deposit intensity; signal attaches to location/type
NIH activity and chronicity indices (Section 5)	Activity index predicts response and flare	Chronicity index predicts; activity index as a whole generally does not	Chronicity index, ESRD: adjusted HR 1.31 per unit (95% CI 1.23–1.39), n = 1945 [13]; activity index adjusted HR 1.05 (95% CI 1.01–1.09)	Chronicity index yes, repeatedly [13,26,28]; activity index weak or null [153,154,176]
Lupus podocytopathy (Section 6)	High response rate; relapse in about 60%	Generally excellent kidney survival; risk concentrated in the FSGS subtype	No ESKD or death over 5–7 years, n = 98 [36]	NR
Lupus vasculopathy other than TMA (Section 7)	Not reported	Adverse, but largely attributable to concomitant proliferative and chronic injury	Any renal vascular lesion, composite endpoint: adjusted HR 1.92 (95% CI 1.01–3.65), n = 225 [182]; univariable HR 5.49 (95% CI 2.6–11.3), n = 285 [183]	Mostly not, once chronicity is entered [35]
Antiphospholipid-antibody nephropathy (Section 8)	Limited data	Association with ESKD inconsistent across cohorts	Histological APSN vs. pure LN, ESRD: OR 5.8 (95% CI 1.7–19.7), n = 112 [193]	Variable; not reported in the largest series
Thrombotic microangiopathy (Section 9)	Limited data	Consistently adverse in large cohorts; attenuates when baseline severity is modelled	Renal TMA, kidney replacement therapy: multivariable HR 6.37 (95% CI 3.28–12.40), n = 1218 [25]	Yes, in large cohorts [25,201]; attenuates to non-significance in a matched series (3.77 → 2.20) [206]
Repeat and per-protocol biopsy (Section 10)	Activity index at the second biopsy predicts flare	Indices at the second biopsy predict functional loss; first-biopsy indices do not	Activity index > 1 at second biopsy, renal flare: OR 23.1 (95% CI 5.1–103.8), n = 114 [231]; chronicity index at second biopsy, ESKD: HR 1.41 (95% CI 1.09–1.82), n = 92 [46]	Yes, at the second biopsy [46,171]

## Data Availability

No new data were created or analyzed in this study. Data sharing is not applicable to this article.

## Supplementary Materials

The following supporting information can be downloaded at https://www.mdpi.com/article/10.3390/medicina62091653/s1: Table S1: PubMed/MEDLINE search string.

## Abbreviations

SLE	Systemic lupus erythematosus
LN	Lupus nephritis
ISN/RPS	International Society of Nephrology/Renal Pathology Society
WHO	World Health Organization
NIH	National Institutes of Health
AI	Activity index
CI	Chronicity index
IF/TA	Interstitial fibrosis/tubular atrophy
TMA	Thrombotic microangiopathy
APS	antiphospholipid syndrome
APSN	Antiphospholipid-antibody nephropathy
FSGS	Focal segmental glomerulosclerosis
eGFR	Estimated glomerular filtration rate
ESKD	End-stage kidney disease
CKD	Chronic kidney disease
CR/PR	Complete/partial response
CYC	Cyclophosphamide
MMF	Mycophenolate mofetil
AKI	Acute kidney injury
HR	Hazard ratio
OR	Odds ratio
RR	Relative risk
IV-S/IV-G class	IV segmental/global (ISN/RPS)
aPL	Antiphospholipid antibodies
ANCA	Anti-neutrophil cytoplasmic antibodies
anti-dsDNA	Anti-double-stranded DNA antibodies
