# Supplementary material for: Kidney Biopsy and Prognosis in Lupus Nephritis: Histological Predictors of Renal Outcome—A Narrative Review
Source: Medicina (Kaunas). 2026 Aug 28;62(9):1653. doi: 10.3390/medicina62091653 (PMC13609859; doi:10.3390/medicina62091653)
Supplement: Supplementary file 1 [file medicina-62-01653-s001.zip › medicina-4448354-supplementary.pdf]

Supplementary Material — Table S1. PubMed/MEDLINE search string

Database: PubMed/MEDLINE. Search executed from inception to 30 June 2026 (publication date). The query below was used to identify primary studies relating lupus nephritis histopathology to renal outcome; records were managed in a reference manager (Zotero) and screened as described in the Methods. It returned 5,391 records (5,357 after removal of 34 duplicates). *Reviews, editorials and letters and non-English-language articles were not filtered in the search string; they were excluded during title/abstract and full-text screening.*

```
("Lupus Nephritis"[Mesh]
 OR "lupus nephritis"[tiab]
 OR ("systemic lupus erythematosus"[tiab] AND nephritis[tiab])
 OR ("SLE"[tiab] AND nephritis[tiab]))
AND
("Biopsy"[Mesh]
 OR biops*[tiab] OR histolog*[tiab] OR patholog*[tiab] OR morpholog*[tiab]
 OR "renal pathology"[tiab] OR "kidney pathology"[tiab]
 OR classification[tiab] OR class[tiab] OR classes[tiab]
 OR "activity index"[tiab] OR "chronicity index"[tiab]
 OR vascular[tiab] OR vascul*[tiab] OR microangiopath*[tiab]
 OR thrombotic[tiab] OR lesion*[tiab] OR involv*[tiab])
AND
(prognos*[tiab] OR predict*[tiab] OR outcome*[tiab]
 OR "renal outcome"[tiab] OR "kidney outcome"[tiab] OR endpoint*[tiab]
 OR follow-up[tiab] OR "follow up"[tiab] OR progression[tiab]
 OR deterioration[tiab] OR impairment[tiab]
 OR remission[tiab] OR relapse[tiab] OR flare[tiab] OR response[tiab]
 OR survival[tiab] OR "renal survival"[tiab] OR "kidney survival"[tiab]
 OR "renal function"[tiab] OR "kidney function"[tiab]
 OR "renal failure"[tiab] OR "kidney failure"[tiab]
 OR "end-stage kidney disease"[tiab] OR "end stage kidney disease"[tiab]
 OR ESKD[tiab] OR ESRD[tiab]
 OR creatinine[tiab] OR proteinuria[tiab] OR eGFR[tiab] OR GFR[tiab])
```
